# A scoping review on determinants of unmet need for family planning among women of reproductive age in low and middle income countries

**DOI:** 10.1186/s12905-015-0281-3

**Published:** 2016-01-15

**Authors:** Joseph K. Wulifan, Stephan Brenner, Albrecht Jahn, Manuela De Allegri

**Affiliations:** 1 Institute of Public Health, Ruprecht-Karls-University, Im Neuenheimer Feld 324, 69120 Heidelberg, Germany; 2 School of Business & Law, Department of Administration & Management Studies, University for Development Studies, P.O. Box UPW 36, Wa, Ghana

**Keywords:** Unmet need, Contraception use, Family planning, Birth spacing and limiting

## Abstract

**Background:**

Poor access and low contraceptive prevalence are common to many Low- and Middle-Income Countries (LMICs). Unmet need for family planning (FP), defined as the proportion of women wishing to limit or postpone child birth, but not using contraception, has been central to reproductive health efforts for decades and still remains relevant for most policy makers and FP programs in LMICs. There is still a lag in contraceptive uptake across regions resulting in high unmet need due to various socioeconomic and cultural factors. In this mixed method scoping review we analyzed quantitative, qualitative and mixed method studies to summarize those factors influencing unmet need among women in LMICs.

**Methods:**

We conducted our scoping review by employing mixed method approach. We included studies applying quantitative and qualitative methods retrieved from online data bases (PubMed, JSTOR, and Google Scholar). We also reviewed the indexes of journals specific to the field of reproductive health by using a set of keywords related to unmet contraception need, and non-contraception use in LMICs.

**Results:**

We retrieved 283 articles and retained 34 articles meeting our inclusion criteria. Of these, 26 were quantitative studies and 8 qualitative studies. We found unmet need for FP to range between 20 % and 58 % in most studies. Woman’s age was negatively associated with total unmet need for FP, meaning as women get older the unmet need for FP decreases. The number of children was found to be a positively associated determinant for a woman’s total unmet need. Also, woman’s level of education was negatively associated – as a woman’s education improves, her total unmet need decreases. Frequently reported reasons for non-contraception use were opposition from husband or husbands fear of infidelity, as well as woman’s fear of side effects or other health concerns related to contraceptive methods.

**Conclusion:**

Factors associated with unmet need for FP and non-contraception use were common across different LMIC settings. This suggests that women in LMICs face similar barriers to FP and that it is still necessary for reproductive health programs to identify FP interventions that more specifically tackle unmet need.

**Electronic supplementary material:**

The online version of this article (doi:10.1186/s12905-015-0281-3) contains supplementary material, which is available to authorized users.

## Background

Many women of reproductive age in low- and middle-income countries (LMICs) would like to avoid or postpone getting pregnant, but are not able to use any family planning (FP) methods [1, 2]. These women face an unsatisfied demand for contraception, which is commonly referred to as *unmet need for FP*. Unmet need for FP is further defined as: *unmet need for limiting*, i.e. any unwanted pregnancy in a woman of reproductive age who does not wish to have any more children; and *unmet need for spacing*, i.e. any mistimed pregnancy in any woman of reproductive age who wishes to delay the birth of her next child by at least two years [3, 4].

National estimates for total unmet need for FP, unmet need for spacing, and unmet need for limiting are usually derived from standardized household-based surveys like the Demographic Health Survey (DHS) [5, 6]. However, the definition of the estimated measurements changed over the past decades. Thus direct comparisons of estimates of unmet need for FP between time periods is not necessarily straightforward [6]. The main difference between the ‘original’ (in use between 2003 and 2008) and ‘revised’ definition (in use after 2008) is how certain sub-groups of pregnant, postpartum amenorrheic and infertile women are classified. In the original definition, unmet need for FP contraceptive calendars (i.e. is a month-by-month retrospective history of births, pregnancies, terminations, and episodes of contraceptive use in the five years preceding the interview) were used to distinguish unmet need from met need (i.e. either unwanted pregnancies in spite of contraceptive use or absence of pregnancies due to continued FP use) and from no need (i.e. absence of a pregnancy due to infertility [6]. In the revised definition contraceptive calendars are no longer in use and the unmet need category was broadened to include all pregnant and postpartum amenorrheic women with an unwanted pregnancy (i.e. regardless of contraceptive behavior at the time). As a result, estimates for total unmet need for FP, for spacing, and for limiting increase slightly when based on the revised compared to the original definition [6].

In many LMIC settings official reproductive health policies and FP programs had started already by the early 1960s [7]. Although in most LMICs, contraceptive knowledge increased over the last few decades, many women continue to have unmet need for FP as result of various demographic and socioeconomic factors [8–11]. Therefore, many LMICs, particularly in Asia, the Middle East, the Caribbean, and Sub-Saharan Africa, continue to show relatively high fertility rates as a result of high unmet need for both limiting and spacing among women of reproductive age [12, 13]. Worldwide, in spite of existing national FP programs, about one in three women who desires to space (16 %) or limit (13 %) the birth of an additional child is still restricted to contraceptive use [7, 14–16].

The concept of unmet need for FP is central to reproductive health policies and research [17, 18]. Especially in LMICs, high unmet need for FP is a main cause for unintended pregnancies (i.e. pregnancies which are mistimed or unwanted at the time of conception), closely spaced births, childbearing at very early age, or clandestine/illegal abortions—all of which are considered main contributors to high maternal and infant mortalities [13, 17, 18]. Unmet need for FP further contributes to high fertility rates leading to rapid population growth [2, 3]. In addition, as unmet need for FP is closely related to high female illiteracy, gender inequality, and poverty, this unsatisfied demand for FP does not only negatively impact women’s reproductive health, but also their ability to participate in economic and educational activities necessary to overcome the cycle of poverty and ill-health [19–21].

For all of these reasons, addressing unmet need for FP has become a key global health priority, tackled by the Millennium Development Goals 4 (reduction of child mortality) and 5 (improvements in maternal health through universal access to sexual and reproductive rights) [22–24]. Although progress has been made towards FP knowledge in LMICs [7, 25], tackling persistent unmet need and low contraceptive use remains a challenge given that consistent adherence and access to FP methods is under influence of a number of different factors [7].

Our scoping review thus aimed specifically at exploring these different factors influencing unmet need for FP in LMICs. The need for this review stems from the fact that available evidence has not yet been systematically appraised and condensed across these settings. Our study is set against the awareness that unmet need for FP differs greatly both between countries and within countries, suggesting that there are always some women with better access than others to contraceptive use [2, 7, 16]. Understanding what factors act as barriers to FP use is essential in establishing an adequate evidence base to support the design of policies aimed at counteracting unmet need. Furthermore, condensing evidence across settings allows a meaningful comparison of experiences and facilitates knowledge transfer across countries. Through the inclusion of both quantitative and qualitative studies, our review aimed both at quantifying the magnitude of unmet need and its influencing factors and at explaining the broader set of factors/elements which influence such unmet need.

## Methods

Scoping studies allow researchers to review the sources and types of existing evidence related to a specific research area in sufficient detail to understand the current status of knowledge related to a scientific topic [26]. “Scoping” refers to the method of mapping, charting, and summarizing existing evidence taken from different published sources to gain a sufficiently comprehensive understanding of a given field of study [26]. The overall focus is on appraising the overall body of evidence on a given topic, with a focus on width rather than depth. We conducted our scoping review according to the ‘York methodology’ described by Arksey & O’Malley [26], but further complemented it with elements of Pluye et al.’s [31] framework for mixed method reviews. This allowed us not only to enrich the review by including quantitative, qualitative, and mixed methods studies, but also to appraise and condense evidence across study types into one single interpretation.

Hereafter, we describe our methodological approach in detail according to the York framework [26].

### Step 1: Identification of research question

As indicated above, our main objective was to appraise evidence available on factors affecting unmet need for FP among women living in LMICs. More specifically, we wanted to understand whether such factors differed depending on the type of unmet need considered, i.e. limiting vs. spacing. Our primary research question was thus framed as: *What are the determinants of unmet need for FP among women of reproductive age in LMICs?* And the subsequent secondary research questions: *What are the determinants of unmet need for limiting?* And: *What are the determinants of unmet need for spacing?*

### Step 2: Identification of relevant studies

To address the research question, we identified the following search terms: “birth spacing”, “birth limiting”, “unmet contraception need”, “unmet need for family planning”, “gap in family planning”, “couple unmet need”, “unsatisfied fertility”, “unattained fertility”, “unmet trend”, and “non-contraception use”. These search terms were further matched with the following: “Sub-Saharan Africa”, “Latin America”, “Asia”, and “Middle East” to specifically include only studies relevant to the LIMCs. As non-contraceptive use and unmet need for FP are considered two sides of the same coin, our search strategy included terms pertaining to both concepts. Using these search terms, we systematically searched relevant electronic databases (PubMed; JSTOR; Google Scholar) for quantitative, qualitative, and mixed methods studies. We limited our search to peer-reviewed studies. Moreover, we searched reference lists of all retrieved studies to identify potentially additional studies matching our search strategy.

### Step 3: Selection of studies for review

We retained only studies published in English between 1980 and 2014, to reflect the period during which official FP policies came into place in most LMICs [23, 27]. We included studies targeting both women and/or men of reproductive ages, as these represent the target population for most FP programs. We excluded studies with main focus on traditional forms of contraception or use of modern contraception for purposes other than FP (i.e. sexual abstinence, pregnancy breastfeeding, HIV/STD prevention, postmenopausal hormone therapy). Table 1 illustrates the selection criteria that informed the study selection process.

**Table 1:** Inclusion/exclusion criteria

Criteria	Inclusion	Exclusion
Study design	A quantitative, qualitative, and mixed method designs.	
Location	Low and middle income countries. (Sub-Saharan Africa, Latin America, Caribbean, Middle East and Asia)	High income countries (with Human Development Index 0.70 and above).
Date	1980 to 2014	Before 1980
Language	English.	Any other language.
Age	Female: 15-49 years.	Female: <15 and >50 years
	Male: 18-54 years.	Male: <18 years and > 54 years.
Research Focus	Main focus on non-use or discontinuation of contraception, as well as unmet need for family planning	Main focus on pregnancy, abstinence, age of sexual debut, number of sexual partners, HIV/STD prevention, use of contraception without consideration of non-use.

### Step 4: Charting of key information

We sorted the information of the selected studies according to the following categories: ‘authors’, ‘year of publication’,’ study location’, ‘main study objective’, ‘study design’ (i.e. study population, survey type, year of data collection), and ‘methodological approach used in data analysis’. Information provided in quantitative studies was further extracted into ‘explanatory variables used’ and ‘statistical associations with outcome measures’; information from review studies were further summarized as ‘key findings’; in qualitative studies we charted the’main themes’ and the ‘relationships between thematic findings’ instead. For all quantitative studies we also charted the prevalence estimates for unmet need for FP (separated into limiting and spacing when available). In line with the focus of our research question, we mapped all statistical associations between the explanatory and the outcome variables used in a study. For qualitative studies, we mapped the main themes reported by the respective authors and how they were found to explain the use/non-use of contraceptives and/or unmet need for FP in a given context. All extracted information was mapped in data charting forms by the first and second author. Charting forms reflected the study typology (quantitative studies specific to one single country, multi-country quantitative studies, and qualitative studies). Charting forms 1 to 4 are included in Additional file 1: Tables S1–S4. The information extracted from the articles into these charting forms constituted the basis for our analysis.

### Step 5: Collating and summarizing of results

In the process of synthetizing the findings for this scoping review, each author repeatedly reviewed the extracted evidence independently. To enhance the validity of the review, individually appraised findings were later triangulated among authors. We first analyzed quantitative and qualitative information separately. To do so, we collated quantitative key findings across studies based on the measures of association between determinants (i.e. explanatory variables suggested by the different authors) and unmet need for FP (i.e. outcome variable). We first compared across all quantitative studies how frequently different explanatory variables for unmet need for FP were used and how often these variables were found to represent a significant determinant for unmet need. We summarized the determinants that resulted in statistically significant associations – either positive or negative – with the total unmet need for FP, the unmet need for spacing, and the unmet need for limiting. We then compared these associations to the country-specific prevalence of unmet need of FP reported at the time of data collection for each study. Qualitative information was organized in form of the main themes identified and explored across the selected qualitative studies [28–30]. Content analysis of these main themes was used to further identify and summarize any contextual factors (e.g. cultural, gender-related, experiential, etc.) that were found to be contributing to the unmet need for FP in a given setting. In a second step, we appraised the qualitative and quantitative information by looking for convergence across studies using different methodological approaches [26, 31, 32].

## Results

The overall information extracted from each study can be reviewed in the charting forms contained in Additional file 1. In the results section we only present a condensed summary of this extracted information as it pertains to the overall research question to be answered by this scoping review. Table 2 provides a summary of quantitative evidence in respect to various determinants of total unmet need; Table 3 provides a summary of quantitative evidence in respect to various determinants of unmet need for spacing and limiting; Table 4 provides a summary of qualitative evidence in respect to various determinants of unmet need for FP. In the text, we present quantitative and qualitative findings of our review together by adopting the pathways framework suggested by Shaikh et al. 2010 [15] to differentiate along the following levels: individual woman (user), partner level, couple level, household and community level, and health service level. Occasionally, additional information taken from the reviewed studies is reported to contextualize the findings summarized in Tables 2 and 3. The source publication of such information is referenced and further details on this information can be reviewed in the charting tables contained in Additional file 1.

**Table 2:** Summary of commonly studied determinants of total unmet need for family planning

Level health service	Availability long-acting FP methods														–
	Access to FP services					–			n					–	–
	Access to FP information		–			n								–	
Level household or community	Rural residence		+	n		+		n		+			+	–	
	Low socioeconomic status		+		n			+	n				+	n	
	Preference for male offspring				–	n						–			
Level couple	Couple discussing FP									n			–	+	
Level partner	Partner’s level of education			–	n	n		n		n		n	–		
	Partner’s desire for more children							–					n		
Level woman	Decision-making authority													–	
	Being religious (M = Muslim, C = Catholic)				+^M^	n	n		–			n	+^C^		
	Woman’s level of own income		–	–		n		–		n		n			
	Woman’s level of education	n	–	–	n	–		–	–	n	n	n	–	+	
	Woman’s awareness or knowledge of FP					–			n	n					
	Previous abortions											+			
	Number of previous children or pregnancies	n	+	n	+	n	+	–	n		+	+	+	+	
	Age of woman at marriage			n	+	+		–				n			
	Age of woman		–	n	–	n	n	±ª	n		±^b^	n	–	–	
	Total unmet need (%)	59	24^r^	45^r^	25	37^r^	21	29	42^r^	52^r^	22	7^r^	41	27	31^r^
	Country or Region	Nigeria	Pakistan	Sudan	Nepal	Ethiopia	Nigeria	Zambia	Ghana	Ethiopia	Nepal	Egypt	Uganda	Eritrea	SSA
	Publication*	Adeyemi et al. 2005 [10]	Ahmed et al., 2011 [48]	Ali and Okud, 2013 [36]	Bhandari et al., 2006 [45]	Hailemariam et Haddis, 2011 [8]	Igwegbe et al., 2009 [11]	Imasiku et al., 2014 [33]	Machiyama et Cleland, 2013 [46]	Mekonnen et Worku, 2011 [12]	Paudel et al. 2011 [34]	Sultan et al., 2010 [42]	Wablembo et al., 2011 [47]	Woldemicael et Beaujot, 2011 [43]	Jain et al. 2014 [64]

+ = statistically significant positive association; – = statistically significant negative association; n = no statistically significant association

* Year of data used for reported prevalence estimation does not necessarily concur with year of publication

^a^ Women’s age positively associated in age range <34 years, negatively associated in age range >34 years

^b^ Women’s age positively associated in age range <30 years, negatively associated in age range >30 years

^r^ estimates for unmet need based on ‘revised definition’ and thus likely overestimating total unmet need for FP compared to ‘original definition’

^C^ Catholic Second

^M^ Muslim

^SSA^ Sub-Saharan Africa

**Table 3:** Summary of commonly studied determinants of unmet need for spacing and for limiting (+ = positive statistically significant association; – = negative statistically significant association; n = no statistically significant association)

Level health service	Access to FP services			–				–
				–				–
	Access to FP information	–						–
		–					–	–
Level household	Rural residence	+	n	+	n	n		–
		+		+	n	+		n
	Low socioeconomic status	+			+			+
		+			+			n
Level couple	Couple discussing FP							+
						n	–	+
Level partner	Partner’s approval of FP							
						n	–	
	Partner’s level of education		–	n	n			
				n	n	n		
	Partner’s desire for more children				–			
					–		+	
Level woman	Decision-making authority							–
								–
	Woman’s approval of FP							
							–	
	Woman’s level of own income	–	n	n	–			
		n		n	–	n		
	Woman’s level of education	n	–	–	–			n
		–		–	–	n	–	+
	Woman’s awareness or knowledge of FP			–				
				n		n	–	
	Experience of child death		n	n				
				–		n		
	Number of previous children or pregnancies	–		n	–			+
		+		+	n			+
	Age of woman at marriage		–	+	n			
				n	–			
	Age of woman	–	n	–	±^a^			–
		–		n	+			n
	Unmet need for limiting (%)	14^r^	14^r^	13^r^	-	13^r^	16	6
	Unmet need for spacing (%)	10^r^	31^r^	24^r^	-	39^r^	42	21
	Country	Pakistan	Sudan	Ethiopia	Zambia	Ethiopia	Rwanda	Eritrea
	Publication*	Ahmed et al., 2011 [48]	Ali and Okud, 2013 [36]	Hailemariam and Haddis, 2011 [8]	Imasiku et al., 2014 [33]	Mekonnen and Worku, 2011 [12]	Ndaruhuye et al., 2009 [37]	Woldemicael and Beaujot, 2011 [43]

* Year of data used for reported prevalence estimation does not necessarily concur with year of publication

^**+**^Top line: associations for unmet need for spacing; bottom lines: associations for unmet need for limiting

^a^ Women’s age positively associated in age range <34 years, negatively associated in age range >34 years

^r^ estimates for unmet need based on ‘revised definition’ and thus likely overestimating unmet need for spacing and limiting compared to ‘original definition

**Table 4:** Summary of explored themes on reasons contributing to the unmet need for FP identified in qualitative studies (+ = contributing; – = not contributing)

Level health service	Lack of trust in FP service provider		+		+		+	+	+
	Unavailability of information on methods		+	+	+	+	+	+	+
	Unavailability of modern methods		+		+		+	+	
Level household or community	Opposition from family	+	+	+					+
	Opposition from community leaders								+
Level couple	Lack of couple discussing FP	+		+	+	+			+
Level partner	Partner’s fear of infidelity/promiscuity	+	+	+	+	+	+		+
	Limited male involvement in FP issues				+	+			+
Level woman	Religious belief	+	+	+	+		+		+
	Desire to space	+	+			+			+
	Reproductive obligation	+	+	+	+	+			
	Woman’s level of education		+						
	Misconception on FP method		+	+	+	+	+		+
	Fear of domestic violence	+							
	Fear of stigma	+		+			+		+
	Fear of side effects		+		+	+			
	Lack of autonomy in decision making	+			+	+	+		
	Country	Ghana	India	Uganda	Uganda	Tanzania	Tanzania	Ethiopia	Guatemala
	Publication	Bawah et al. 1999 [39]	Hall et al., 2008 [38]	Kabagenyi et al., 2014 [19]	Kaida et al., 2005 [40]	Mosha et al., 2013 [21]	Plummer et al., 2006 [41]	Sonalkar et al., 2013 [50]	Ward et al., 1992 [35]

**Fig. 1:**
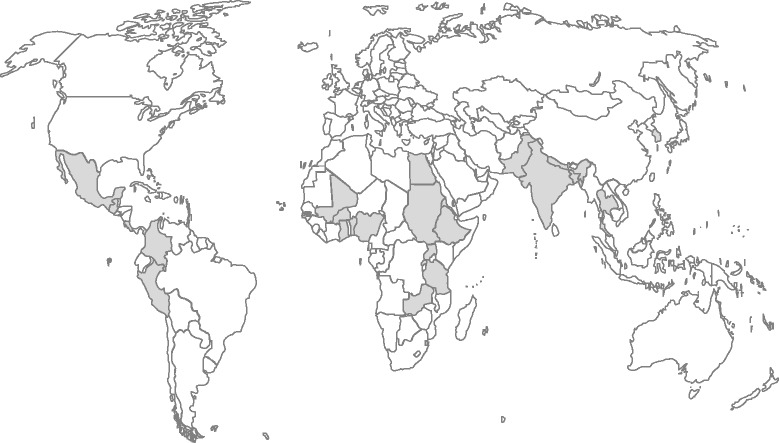
Geographic distribution of reviewed studies. This map provides an overview of the geographic distribution (shaded countries) of those studies in this review with a country-specific focus. (Source: Author’s construct using the World map free template.net, 2015) [66]

Of the 283 articles initially identified by our search criteria, only 34 studies met the inclusion criteria for our review. Of these, twenty one were quantitative studies with a single country focus, five were quantitative studies with a multi-country or regional focus and eight were qualitative studies. Sixteen studies were published within the past five years (2010 onwards), fourteen studies between 2000 and 2010, while four studies were published before the year 2000. In terms of geographical distribution, eleven studies were specific to countries in Sub-Saharan Africa, seven to countries in Asia, two to countries in Northern Africa and one in Latin America; five studies covered multiple low income countries, either in general or by global regions. See Fig. 1 for the geographical distribution of reviewed studies with country-specific focus.

Among the eight qualitative studies, six were conducted in a SSA setting, one in India, and one in Guatemala. Five studies interviewed both women and men of different ages and marital status, one study only interviewed women, one study interviewed adolescents, and five studies included different opinion leaders and FP stakeholders (e.g. community leaders, public and private FP providers). Seven studies used either focus group discussions or key informant interviews or both, while one study used a participant observation approach (i.e. in-depth assessment of attitudes towards FP methods within a community in which the researcher is part of during the study period).

Among the 26 quantitative studies, the majority relied on cross-sectional household-based data while the remaining studies relied on facility-based survey data. This variable use of data sources further limited direct comparison across all studies. Ten studies differentiated the outcome variable into unmet need for limiting and unmet need for spacing. Of the twenty six quantitative studies published in or after 2003 (i.e. after introduction of the original definition of unmet need for FP), 11 studies based their estimates of unmet need for FP on the “original” definition of unmet need.

Among the country-specific studies included in this review, highest estimates for total unmet need for FP were found in Nigeria (59 % in a facility-based survey from 2004 in Ife-Ife teaching hospital) and in Rwanda (58 % based on DHS data from 2005). The highest estimate for the unmet need for spacing of 42 % was also reported for Rwanda in 2005, while the highest estimate for the unmet need for limiting was reported with 22 % in Egypt in 1997 (both based on DHS data). Lowest estimates for total unmet need for FP were found in Egypt in a household-based survey in Eastern Cairo (7 % in 2009), in India in a household-based survey in Haryana state (18 % between 2003 and 2005), and in Nigeria in a facility-based survey at Nnewi teaching hospital (21 % between 2008 and 2009). Among studies with a regional focus, highest estimates for total unmet need for FP were found for the SSA and Asian regions with 74 % and 62 %, respectively, in a review of several DHS studies form the mid-1990s.

Tables 2 and 3 summarize the associations reported across studies between different factors (i.e. explanatory variables tested by each study) and the unmet need outcome measures: total unmet need for FP (Table 2), and unmet need for spacing and for limiting (Table 3). The tables outline the direction of a statistically significant association together with some information on the respective study context (authors, publication year, study country/region, estimates of unmet need corresponding at time of data collection). Table 4 provides an overview on different themes emerged from the reviewed qualitative studies addressing factors related to unmet need for FP. This combined evidence is reported in the following sections.

### Individual woman level

*Age of the woman* was explored in eleven quantitative studies. In only six of these studies, age as a determinant of unmet need was found to be significant. If significant, a woman’s age was negatively associated with total unmet need for FP, meaning as women get older the unmet need for FP decreases. Two studies—one from Zambia [33] and one from Nepal [34] – indicated that the relationship between a woman’s age and her unmet need changed across the reproductive age range between younger and older reproductive years. While unmet need increased with the years of life below the age of 34 years, it decreased with age once a woman reached 34 years or more. A woman’s age was also negatively associated with the unmet need for spacing, meaning that as a woman gets older the unmet need to space pregnancies decreases. In a study from Zambia [33] this association was again positive below the age of 34 years and became negative thereafter. In contrast, qualitative evidence in a study from Guatemala highlighted how older women (35–40 years of age) who have already born several children expressed a higher need for spacing methods [35]. These Guatemalan women felt that additional children have a negative impact on older women’s health and jeopardize their financial ability to feed and care for already existing children. Age was found to be less of a determinant for the unmet need for limiting, except in the Zambian study [33] where a positive association between age and the need for limiting was detected. Besides its direct effect on unmet need, a woman’s age also influenced other determinants of unmet need, such as discussions on FP. Older women from Rwanda and Eastern Sudan, for example, seemed to be more likely to engage in discussions on contraceptive use with their partners compared to their younger counterparts [36, 37]. In line with this, although younger women were clearly in favor of birth spacing rather than limiting and reversible rather than permanent methods of FP [35, 38], they still were often less knowledgeable in modern FP options [35] and less likely to engage in FP discussion with health workers compared to older women [36, 37]. This reluctance in actively expressing their FP needs is in parts explained by prevailing stigma attached to contraceptive use in young females, such as unfaithfulness and extramarital relations [19, 39, 40], resistance to and intervening with God’s reproductive plans [35], or distrust in health workers confidentiality [41].

*Number of previous children or pregnancies* as a determinant for unmet need was explored in twelve quantitative studies. In eight of these studies significant associations with unmet need were identified. In seven studies, the number of previous children or pregnancies was found to be a positively associated with a woman’s total unmet need for FP, meaning as the number of children/pregnancies increased, so did the total unmet need. One study from Zambia reported a negative association [33]. The number of previous children or pregnancies was mostly positively associated with the unmet need for limiting, while the effect on unmet need for spacing was less consistent across studies. Evidence from qualitative studies (Table 4) indicated certain cultural norms towards females’ reproductive obligations, which was reported in six of the reviewed qualitative studies, and thus representing dominant contributors to the unmet desire for birth limitation. Strong patriarchal traditions, higher value for male over female children, and mainly rural practices ensuring economic or social security based on large family sizes expect women to bear many children [19, 21, 38–40].

Only one study from Egypt [42] identified the *number of previous abortions* as a significant determinant increasing total unmet need for FP. Other variables at the individual woman level, such as *age at marriage* or the *experience of child death*, varied in their significance patterns between studies.

Twelve quantitative studies examined a *woman’s level of education* as possible determinant of unmet need, which was found to have a significant association in seven studies. Six of these studies showed that a woman’s higher level of education is associated with a decrease in total unmet need, unmet need for limiting, and unmet need for spacing. One study from Eritrea [43] shows that, higher educational levels were associated with increased unmet need for limiting. In general, women’s education seems to have a stronger effect than partner’s education on contraceptive use [44]. Qualitative evidence in respect to the role of female education on unmet need is relatively meager and was only identified in one of the qualitative studies (Table 4). Qualitative interview findings from India [38] however describe how positive attitudes towards modern forms of contraception and a clear understanding of actual side effects related to certain FP methods are more common among women with higher educational standing.

A *woman’s decision-making authority* was only examined in one quantitative study and found to decrease both unmet need for spacing and limiting in the Eritrea study (see Table 3) [43]. Three of the reviewed qualitative studies explored the role of female decision-making autonomy (Table 4) and found that especially in patriarchal settings where men are sole decision-makers [21] or wives considered male property [39], women are expected to simply execute their husbands’ ideals of reproduction. Nevertheless, even in contexts of restrictive gender norms, women reported specific coping strategies in order to maintain some level of sexual autonomy, such as the secret use of contraception [21, 40] or forms of evading sexual contacts that are socially acceptable [39]. Based on the quantitative evidence in Tables 2 and 3 it seems, however, that a woman’s independent work or income status, and thus a more disposable income and economic decision-making power, also allows for more reproductive autonomy [33, 36, 37, 43, 44].

A *woman’s approval of FP* was only examined in one quantitative study in Rwanda with a focus on unmet need for limiting only (see Table 3). Based on this Rwandan study [37] a woman’s approval of FP seemed to decrease her unmet need for limiting. Qualitative evidence in Table 4 indicates that female approval or disapproval of modern contraceptive methods is mainly influenced by women’s misconceptions of FP methods (identified in six studies) and/or limited understanding of potential side effects (identified in three studies). Fears of side effects are often rooted in an overestimation of rare complications (e.g. permanent infertility as result of OCP use, malignancies or even death as result of hormonal contraceptives or IUDs), wrong indications (e.g. abuse of OCP without physician guidance), or based on non-validated rumors (e.g. commercial condoms to be purposefully infected with HIV or only used in order to prevent SDIs) [19, 21, 35, 38, 40, 41].

The role of a woman’s *awareness* or *knowledge of FP* was examined in three studies and found to be negatively associated either with total unmet need (Table 2), with spacing, or with limiting (Table 3). While the significance of this determinant in relation to unmet need was relatively vague across quantitative studies, the qualitative evidence was much clearer (Table 4). Both FP knowledge and knowledge transfer were found to be very poor – both for traditional and modern forms of contraception – and more limited among men compared to women [35, 38, 40, 41]. Especially in settings where FP use is stigmatized (identified in four studies), sharing of FP knowledge between women is limited. In two studies, for example, interviewed women initially denied any contraception use, but once asked in a more private setting, respondents confirmed at least some use of FP. These women explained their behavior based on the fact that discussing and receiving information on FP practices from fellow women carries some social risks in the form of stigma [38, 41].

The influence of *religious beliefs* on FP behavior appears to be more complex. Among six quantitative studies examining the role of a woman’s religious belief, significant associations with unmet need for FP were found in only three studies (Table 2): in a Nepal study, Muslim belief was strongly associated with an increase in unmet need when compared to Hinduism [45]; in a study in Ghana [46], Catholicism was strongly associated with a decrease in unmet need when compared to other religious beliefs; and in an Ugandan study [47] this association was positive. A qualitative study from Uganda, supported this finding by identifying Anglicans to be more open towards contraceptive use compared to Muslims and Catholics [40]. Overall, as shown in Table 4, there was more qualitative evidence on the role of religious beliefs on unmet FP needs (identified in six studies). In a study in India for example, Hindu traditions includes a number of menstrual taboos that require close observation of a woman’s fertility and thus make contraceptive use very difficult [38]. In two studies from Uganda and Tanzania, religious and traditional beliefs consider it a duty for couple to reproduce to follow a divine directive with contraceptive use not having any moral legitimacy [19, 41]. The influence Christian churches can have on reproductive needs was strongest in a Guatemalan study, where contraceptive use is associated with mortal sin and churches ensure that young people and couples do not receive adequate information on modern FP methods [35].

### Partner level

The partner’s contribution to unmet need was examined in seven quantitative studies (Table 2). Particularly the role of *partner’s education* was found to be a significant negative determinant for unmet need in two of these studies. Two of these seven studies further examined the *partner’s desire for additional children*, which was found significantly associated with unmet need in only one study from Zambia. Of seven reviewed qualitative studies in Table 4, all identified men as often being hesitant to approve of any contraceptive use, mainly out of fear of losing their role as family heads and/or of indirectly encouraging their wives to be unfaithful or promiscuous [21, 38–40]. Three studies further mentioned that men had only limited knowledge on most modern contraceptive methods and it was felt that FP programs tend to target mainly women [21, 35, 40].

### Couple level

At the couple level, *couple’s active discussion of FP* was examined in only four of the quantitative studies (Tables 2 and 3) and found to be a significant determinant of total unmet need, spacing, or limiting in three studies. While in Uganda a decrease in total unmet need as result of couple discussion was seen [47], in Eritrea an increase in unmet need for spacing and limiting could be shown [43], while in the Rwandan study [37] a decrease in the unmet need for limiting was evident. Five qualitative studies explored the role of couple discussion and demonstrated that communication between husband and wife actively shape decisions on contraception use, especially in relation to the overall number of desired children, and thus affect the perception of unmet need [38, 39]. Still, interviews across settings revealed that little or no discussion occurs among partners, often because contraceptive use is considered “a woman’s domain”, especially in rural settings with strictly divided gender roles [19, 21, 40].

### Household or community level

Low socioeconomic status and/or rural residence were examined by nine of the reviewed quantitative studies and both found to increase total unmet need for FP in five studies [8, 12, 33, 47, 48], while in the other studies no such association could be found [36, 43, 45, 46] (Table 2). *Low socioeconomic status* significantly increased unmet need for spacing in the studies from Pakistan, Zambia, and Eritrea [33, 43, 48] (Table 3), mainly as women from lower socioeconomic status are very likely to have lower educational level [38]. *Rural residence* increased both unmet need for spacing and limiting in Pakistan [48] and in Ethiopia [8]. One study from Eritrea [43], however, found a decrease in the unmet need for spacing as a result of rural residence, while in Ethiopia [12] a positive relationship between rural residence and unmet need for limiting, but not for spacing, was observed. The influence of household economics was explored in four qualitative studies identifying the role of opposition from family members or community leaders to FP as potential determinant of unmet need (Table 4). Men in some rural settings, for example, considered birth control to negatively affect the productivity of a household, on the one hand due to reduced family size in households depending on agricultural income and on the other hand as women experiencing side effects from using contraception incur additional medical expenses to households [19]. Women, especially in urban settings, instead considered FP supportive to household economics, as the costs of child rearing (e.g. nutrition, education, medical) could be better allocated and controlled [21, 38]. Furthermore, across studies a variety of additional determinants could be identified at the household level to affect unmet need for FP. In Nepal and Egypt the families’ preference for male offspring showed a negative association – meaning that the total unmet need for FP decreased in settings where higher value was given to a woman bearing sons [42, 45]. Some qualitative studies suggested limited contraceptive use in communities where high value is given to many children might increase unmet need [21, 35, 38, 40, 41]. In India, women described how FP use and women’s fertility was closely observed by family members (in particular mothers-in-law) [38]. Also in societies where bride payments are judged against a woman’s ability to give birth to several children, deep expectations prevail for women’s reproductive role and women’s need for FP [39]. This social pressure attached to women’s desire to control births can in extreme cases even lead to women being subjected to physical abuse and battering if they attempt using contraceptives – even if just for the purpose of delaying a pregnancy [39].

### Health service level

Determinants grouped at the health service level, such as *access to FP information*, *access to FP services*, or the *availability of long-acting FP methods* were examined in five quantitative studies and found to be negatively associated with the total unmet need for FP, but also with both the unmet need for spacing and for limiting (Tables 2 and 3). Health service factors, such as FP provider behavior (friendliness towards clients), quality of care given, user fee payments, and proximity to FP centers were commonly mentioned to influence contraception use [2, 16, 49] (see extraction charts in Additional file 1: Tables S1-S4). The role of health service level determinants was explored in seven qualitative studies (Table 4). In respect to the health service level, the reviewed studies pointed at a variety of factors contributing to unmet need. Five of these studies identified lack of trust towards FP services providers as a contributor to unmet need. From a demand perspective, FP service users criticize to be rudely treated by FP officials [38, 40], to have only limited trust in FP officials’ clinical competency and in keeping their clients’ confidentiality [38, 40], the poor distribution of FP outlets [38, 40, 41], and that FP information is not provided in local languages [35, 40].

All seven studies identified shortcomings in the provision of information on and supplies of modern FP methods as determinants of unmet need. From a programming or supply perspective, the most challenging aspects in providing FP services seems to meet the quantity and quality of FP supplies and information materials, but also to ensure sufficient competency among the service personnel [50]. In addition, frequently voiced criticism, mainly by men, was related to how FP services primarily target women while husbands and partners often feel excluded or their initiatives unappreciated [19, 35, 40]. Taken together, service providers’ incompetence, occasional stock outs of contraceptives, and the inadequacy of a client’s preferred FP method were brought up as common reasons for unmet need for FP [39, 41, 50].

## Discussion

Our review of 37 studies on contraceptive use and unmet need for FP covered multiple LMICs in Africa, Asia, the Middle East, and Latin America. With a few exceptions, the reviewed studies provided evidence for high unmet need for FP across these settings. One the one hand, we were able to detect similarities in of factors and determinants that influence unmet need for FP by comparing evidence across study settings. On the other hand, comparison of determinants across study methodologies, we found that while almost every quantitative study considered women’s age or educational level as relevant factors to be explored, these factors were rarely mentioned in as relevant by the various respondents interviewed in the qualitative studies. Given the almost standardized use explanatory variables in quantitative surveys without any significant relevance (e.g. partner’s educational level) or even contradictory associations (e.g. rural residence) to the unmet need for FP, the reviewed qualitative evidence offered a valuable additional dimension of available evidence. Given this variety of evidence identified by our mixed methods-based review, using Shaikh’s [15] conceptual approach to assessing FP allowed us to structure the available evidence yielded by different study methodologies, study populations, and study contexts more comprehensively.

Among the demographic factors associated with unmet need for FP, the relationship between a woman’s age and the number of living children was most prominent in pointing out how the need for FP changes over the course of women’s reproductive age. Whereas the need for spacing was more likely not to be met among younger women who have not yet achieved their desired fertility goals and hence are more interested in spacing future pregnancies, the unmet need for limiting was stronger during later reproductive years once the desired family size is achieved and no further pregnancies are wanted anymore. These findings are consistent with other studies focusing on women’s fertility choices [51–53]. Considering the variation in unmet need for FP over the reproductive life span, woman’s reproductive needs require to be understood more individually and thus addressed in more differentiated ways. For example, the availability of methods of FP should better reflect the reproductive needs. While younger reproducing women might opt for temporary, less invasive forms of contraception, women in later reproductive age might prefer long-lasting or even permanent forms of birth control. FP policies and programs should therefore target educational messages more specifically to women’s desire for spacing or limiting.

Woman’s education and occupational status (as a proxy of financial autonomy and social status) were also identified as additional important factors influencing whether the need for FP can be easily met. The available evidence suggests that female occupation and an independent income increase a woman’s autonomy by reducing gender inequality and improving financial decision-making within a household [54–57]. The fact that investments in women’s education and autonomous employment can contribute to an increase in the uptake of FP and thus in a reduction in unmet need is suggestive for the need of policies to act beyond the health sector alone.

Furthermore, broad consensus exists that women need to be able to access all necessary knowledge on FP methods, including information on where to obtain the contraceptives of their choice, how to use them correctly, and at what costs [7]. Our review revealed repeatedly that women in LMICs, particularly younger women, lack basic knowledge on reproductive physiology and on modern as well as traditional FP methods. Furthermore, lack of adequate FP knowledge hampers females’ approval of modern contraception and sexual autonomy. Filling these knowledge gaps will require ongoing investments in awareness campaigns of various forms, such as mass media dissemination [54] or peer educational approaches [55, 56]. In addition, while religious belief systems tend to play a fairly central role for most LMIC societies, religious leaders tend to take an often hesitant or even obstructive position towards modern contraception [57, 58]. Winning religious groups or church-based health care providers to support the promotion of FP-centered life-styles might also represent a potential alternative education and information strategy. Furthermore, given the role that husbands and other family members, such as mothers in law, seem to play in in a woman’s decision to use contraceptives, additional programmatic approaches need to be considered that involve and educate men more explicitly on FP aspects and strengthen couples’ and especially women’s ability to decide about their individual reproductive needs [54].

Given the central role that childbearing occupies in most societies, reproductive health programs need to be strategically tailored to address specific cultural or traditional concerns that prevent women in LMICs from taking up FP. Especially health care providers should be able to reduce cultural barriers to some extent in order to adequately address women’s concerns on modern FP methods [59, 60]. The first step towards providing culturally sensitive services includes conveniently located, client-friendly, respectful, and confidential services [61]. Integrating FP services within the delivery of other services could be one possible way to reach more women and reduce gaps in unmet need [62, 63]. For instance, the use of an unobtrusive referral message that linked FP to the Expanded Program of Immunization (EPI) was tested in an operations research study in Togo. The introduction of the referral message was accompanied by an 18 % increase in awareness of available FP services and an increase in the average monthly number of new FP clients of 54 % [63].

Modern FP services are conceptualized as a continuum that covers patient counseling, provision of contraceptives, as well as patient follow ups. Efforts aimed at improving the quality of FP services should be directed not only towards attracting new clients, but also towards preventing contraceptive discontinuity [64]. It is important that FP providers are sufficiently trained in the correct information on FP methods, their side effects, and their application to female clients. The assessment of FP service quality should include the following six elements: choice of method, information given to client, provider competence, provider relation with client, follow up mechanism, and appropriate constellation of services to make contraceptive use comfortable for couple [65]. Women in postpartum, breastfeeding or approaching menopause need to be advised by health providers on the likelihood of becoming pregnant [64].

### Methodological considerations

Compared to systematic reviews, it was not the intention of our mixed method scoping review to assess the methodological quality of the studies included nor to condense evidence across studies in a quantitative manner (through methodologies such as meta-analysis). Our aim was primarily that of collating the overall evidence available to provide a description of the current state of knowledge on the matter at stage. In addition, as outlined earlier, we included only studies published in English. Given the large portion of non-Anglophone countries in SSA and Latin America, we might have missed quite some evidence published in other languages.

## Conclusion

This review demonstrates that there is a noticeable gap regarding awareness and uptake of contraception leading to high unmet need for family planning. Both the factors associated with unmet need and the reasons provided by women to explain their low contraceptive use appear largely similar across settings. This suggests a high potential for knowledge transfer, as countries can learn from one another as they implement strategies to increase contraceptive use and reduce unmet need. We therefore conclude that unmet need for family planning remains high in many LMICs with about one in three women of reproductive age not using contraception in spite of their desire to delay or limit child birth. FP programs aiming at reducing unmet need should therefore have a specific focus on younger women’s need for spacing and older women’s need for limiting. Partners and household members can play various roles in supporting or hampering women’s FP decisions and therefore be more directly reflected in FP program designs. Effective forms of information, education and communication focusing on both women and men seem to indicate a more grounded approach in counteracting persisting unmet need trends in LMICs.

## Additional file

Additional file 1:
**Contains a pdf file with the charting forms prepared during the information extraction and sorting process outlined in Step 4 of the Methods Section.** For each of the selected studies under review, a separate entry was. (PDF 461 kb)
