# Supplementary material for: A scoping review on determinants of unmet need for family planning among women of reproductive age in low and middle income countries
Source: BMC Womens Health. 2016 Jan 15;16:2. doi: 10.1186/s12905-015-0281-3 (PMC4714507; doi:10.1186/s12905-015-0281-3)
Supplement: Additional file 1: — Contains a pdf file with the charting forms prepared during the information extraction and sorting process outlined in Step 4 of the Methods Section. For each of the selected studies under review, a separate entry was. (PDF 461 kb) [file 12905_2015_281_MOESM1_ESM.pdf]

Appendix

Charting Form 1: Mapping of reviewed country-specific quantitative studies on determinants of unmet need for family planning and/or non-contraceptive use in low- and middle-income countries.

Authors, year, location	Study objective	Study design	Analytical approach	Explanatory variables used / Definition of unmet need	Statistical findings / associations with main outcome*	Other important findings
Adeyemi et al. 2005, Nigeria	Prevalence of unmet need among women during the post-partum period.	Cross-sectional facility-based survey of 256 post-partum women between ages 17-40 having attended ANC services at Ife-Ife teaching hospital (data: 2003-04).	Univariate distributions	- Woman's level of education<br>- Parity<br>- Knowledge of FP<br><br>Unmet need estimates based on **original definition** *(i.e. further differentiation of unwanted pregnancies and infecundity based on contraceptive use)*.	**Prevalence:**<br>- Unmet need: 59%<br>- FP use: 41%<br><br>**Association with FP use:**<br>- Level of education woman: n.s.s.<br>- Parity: n.s.s.	**Reasons for FP non-use**<br>- Wide gap between awareness / knowledge and FP use for most modern contraceptive methods<br>- Currently breastfeeding<br>- Health concerns<br>- Currently not sexually active
Ahmed et al. 2011, Pakistan	Determinants of unmet need and contraceptive use among women included in DHS 2006/2007.	Cross sectional household survey (PDHS 2006/2007) in 10,023 married women of reproductive age.	Univariate distributions and multivariate regression analysis	- Woman's age<br>- Woman's level of education.<br>- Residence (rural/urban, province)<br>- Woman's paid work status<br>- Poverty level<br>- Exposure to FP information<br><br>Unmet need estimates based on **revised definition** *(i.e. no differentiation of unwanted pregnancies and infecundity based on contraceptive use)*.	**Prevalence:**<br>- Unmet need: 24% (10% for spacing, 14% for limiting)<br><br>**Association with unmet need (spacing / limiting / total):**<br>- Age woman: (-)/(-)/(-)<br>- Edu level woman: n.s.s./(-)/(-)<br>- Number children: (-)/(+)/(+)<br>- Rural residence: (+)/(+)/(+)<br>- Being poor: (+)/(+)/(+)<br>- Paid work woman: (-)/n.s.s./(-)<br>- FP info access: (-)/(-)/(-)	**Reasons for unmet need**<br>- Opposition by partner or family<br>- Health concerns<br>- Gap between knowledge and service availability
Ali and Okud. 2013, Sudan	Prevalence and determinants of unmet need and demand for FP among women in Kassala State.	Prospective cross-sectional household-based survey of. 812 married women of reproductive age. (data collection: 2012) .	Univariate distributions and multivariate regression analysis	- Woman's age<br>- Woman's age at marriage<br>- Parity<br>- Woman's education<br>- Partner's education<br>- Residence (rural/urban)<br>- Woman's paid work status<br>- Experience of child death<br><br>Unmet need estimates based on **revised definition**.	**Prevalence:**<br>- Unmet need: 45% (31% for spacing, 14% for limiting)<br>- FP use: 26%<br><br>**Association with unmet need (spacing / total):**<br>- Age at marriage: (-)./n.s.s.<br>- Education level woman: (-)/(-)<br>- Education level partner: (-)/(-)<br>- Paid work woman: n.s.s./(-)<br>- All other variables: n.s.s./n.s.s.	

*n.s.s. = not statistically significant association, (+) = positive statistically significant association, (-) = negative statistically significant association

ANC = antenatal care; DHS = Democratic Health Survey; FP = family planning

Authors, year, location	Study objective	Study design	Analytical approach	Explanatory variables used / Definition of unmet need	Statistical findings / associations with main outcome*	Other important findings
Bhandari et al. 2006, Nepal	Prevalence and determinants of unmet need for FP among women in a district in Eastern Nepal.	Cross-sectional household-based survey of 1,076 married and cohabitating women between the ages of 15-44. (data collection: year not mentioned).	Univariate distributions and multivariate regression analysis	- Woman's age<br>- Woman's religion (Muslim vs. Hindu)<br>- Socio-economic status<br>- Woman's education<br>- Partner's education<br>- Family type (nuclear vs. joint)<br>- Woman's age at marriage<br>- Total number of children<br>- Sex of living children (sons vs. daughters)<br><br>Unmet need estimates based on **original definition**.	**Prevalence:**<br>- Unmet need: 25% (10% for spacing, 16% for limiting)<br>- FP use: 49%<br><br>**Association with unmet need:**<br>- Age woman: (-)<br>- Being Muslim: (+)<br>- Age at marriage: (+)<br>- Number of children: (+)<br>- More daughters than sons: (+)<br>- All other variables: n.s.s.	
Casterline et al. 2001, Pakistan	Determinants of unmet need and contraceptive use among married couples in Punjab province.	Cross sectional household-based survey of 1,310 married women between the ages of 20-44 years and 554 of their partners. (data collection: 1996).	Univariate distributions and multivariate regression analysis	- Motivation to use FP<br>- Knowledge/awareness of FP<br>- Woman's acceptability of FP<br>- Partner's attitude towards FP<br>- Adverse health effects<br>- Accessibility of FP services<br><br>**Background variables used in model:**<br>- Intention to use FP in future<br>- Number of living sons<br>- Urban residence<br>- Number of household assets<br>- Woman's level of education<br>- Woman's decision-making capacity<br>- Exposure to FP information<br><br>**Easterlin Synthesis Framework** of unmet need (based on decision/ motivation to avoid pregnancy; cost of preventing pregnancy) closely aligned with **original definition** of unmet need.	**Prevalence:**<br>- Intention to use FP: 35%<br><br>**Association with woman's intention to use FP:**<br>- Awareness/knowledge FP woman: (+)<br>- Acceptability FP woman: (+)<br>- Partner's approval of FP: (+)	**Key obstacles to FP use:**<br>- Perceived social and cultural unacceptability of FP<br>- Wives' perceptions that their partners are opposed to or not supportive of FP<br>- Women's health concerns when using FP<br><br>**Other:**<br>- Discordance between wife's perception of partner's attitude towards FP and partner's acceptance of FP.<br>- Motivation to avoid pregnancy only little influence on intention to use FP

*n.s.s. = not statistically significant association, (+) = positive statistically significant association, (-) = negative statistically significant association

DHS = Democratic Health Survey; FP = family planning

Authors, year, location	Study objective	Study design	Analytical approach	Explanatory variables used	Statistical findings / associations with main outcome*	Other important findings
Casterline et al. 2003, Egypt	Persistence of unmet need for FP and relationship between unmet need and unwanted fertility among women in Upper Egypt.	2-year longitudinal household-based survey (baseline: EDHS 1995) of a cohort of 2,444 married women of reproductive age. (data collection: 1995-97).	Univariate distributions	- Woman's fertility preference<br>- Access to FP services<br>- Quality of FP services<br>- Concerns of adverse effects of FP<br>- Exposure to risk of becoming pregnant<br>- Partner's decision-making capacity regarding FP<br><br>Unmet need estimates based on **original definition**.	**Prevalence:**<br>- **1995:** unmet need: 28% (11% for spacing, 18% for limiting)<br>- **1996:** unmet need: 32% (11% for spacing, 21% for limiting)<br>- **1997:** unmet need: 34% (13% for spacing, 22% for limiting)	**Persistence of unmet need:**<br>- 59% women remained in same FP need category<br>- 34% women shifted from unmet need to no/met need and vice versa (14% vs. 20%)<br>- FP need category as reasonable predictor of intended and unintended fertility<br>- majority (53%) of unintended births in women never having practiced FP
Hailemariam and Haddis. 2011, Ethiopia	Prevalence and determinants of unmet need and demand for FP in Southern Nations.	Data from two cross-sectional household-based surveys (EDHS 2000, 2005) of 2,133 and 1,988 married women between the ages of 15-49.	Univariate distributions and multivariate regression analysis	- Woman's age<br>- Woman's age at marriage<br>- Number of living children<br>- Sex of living children<br>- Experience of child death<br>- Residence (urban / rural)<br>- Woman's level of education<br>- Partner's level of education<br>- Access to FP information<br>- Paid work status<br>- Religion (Christian vs. Muslim)<br>- Knowledge of FP<br>- Access to FP service<br><br>Unmet need estimates based on **revised definition**.	**Prevalence:**<br>- **2000:** unmet need: 35% (24% for spacing, 11% for limiting)<br>- **2005:** unmet need; 37% (24% for spacing, 13% for limiting)<br><br>**Association with unmet need (spacing / limiting / total):**<br>- Age woman: (-)/n.s.s./n.s.s.<br>- Age at marriage: (+)/n.s.s./(+)<br>- Number living children: n.s.s./(+)/n.s.s.<br>- Child death: n.s.s./(-)/|n.s.s.<br>- Rural residence (+)/(+)/(+)<br>- Education level woman: (-)/(-)/(-)<br>- FP knowledge: (-)/n.s.s./(-)<br>- Access to FP: (-)/(-)/(-)<br>- All other variables: n.s.s.	**Demand for FP:**<br>- Knowledge about at least one FP method significantly increases a woman's demand for FP

*n.s.s. = not statistically significant association, (+) = positive statistically significant association, (-) = negative statistically significant association
DHS = Democratic Health Survey; FP = family planning

Authors, year, location	Study objective	Study design	Analytical approach	Explanatory variables used	Statistical findings / associations with main outcome*	Other important findings
Igwegbe et al. 2009, Nigeria	Prevalence and determinants of unmet need for FP among antenatal women in Nnewi.	Cross-sectional facility-based survey of 356 women attending antenatal care services at Teaching Hospital. (data collection: 2008-09).	Univariate distributions and multivariate regression analysis	- Woman's age<br>- Woman's level of education<br>- Woman's religion<br>- Parity<br>- Partner's paid work status<br><br>Unmet need estimates based on **original definition**.	**Prevalence:**<br>- Unmet need: 21% (15% for spacing, 6% for limiting)<br><br>**Association with unmet need:**<br>- Parity: (+)<br>- All other variables: n.s.s.	**Reasons for non-use:**<br>- Partner disapproval (37%)<br>- Health concerns (29%)<br>- Religious beliefs (18%)<br>- Lack of access (16%)<br><br>**Other:**<br>- Most common FP methods known/used: Billings method (64%), male condom (56%).<br>- No significant associations between awareness and use of given FP method (exception: hormone injection).<br>- Main sources of FP info: health workers (66%), radio (37%).<br>- Main benefits of FP: spacing (73%), limiting (41%).
Imasiku et al. 2014, Zambia	Ethnicity as determinant for unmet need for FP and contraceptive use in Zambia.	Cross-sectional household-based survey (ZDHS 2007) of 4,343 married women between the ages of 15-49.	Univariate distributions and multivariate regression analysis	- Woman's ethnicity<br>- Woman's age<br>- Woman's age at marriage<br>- Number of living children<br>- Parity<br>- Residence (rural/urban)<br>- Geographical province<br>- Partner's age<br>- Partner's level of education<br>- Partner's desire for future children<br>- Woman's level of education<br>- Woman's paid work status<br>- Poverty level<br><br>Unmet need estimates based on **original definition**.	**Prevalence:**<br>- Unmet need: 29%<br><br>**Association with unmet need (spacing / limiting / total):**<br>- Age woman: (+)/(+)/(+)<br>- Age at marriage: n.s.s./(-)/(-)<br>- Parity: (-)/n.s.s./(-)<br>- Province not Luapula (-)/(-)/(-)<br>- Education level woman: (-)/(-)(-)<br>- Income woman: (-)/(-)/(-)<br>- Being poor: (+)/(+)/(+)<br>- Partner desires more children: (-)/(-)/(-)<br>- Desire partner for fewer children: n.s.s./n.s.s./(-)<br>- All other variables: n.s.s.	**Ethnicity as determinant of unmet need for FP:**<br>- Ethnicity is not a significant predictor of unmet need for FP in Zambia.

*n.s.s. = not statistically significant association, (+) = positive statistically significant association, (-) = negative statistically significant association

DHS = Democratic Health Survey; FP = family planning

Authors, year, location	Study objective	Study design	Analytical approach	Explanatory variables used	Statistical findings / associations with main outcome*	Other important findings
Jain.1999, Peru	Persistence of unmet need for FP in Nor-Oriental del Maranon and Lima.	2-year longitudinal household-based survey (baseline: DHS 1991/1992) of a cohort of 1,093 married women of reproductive age.(follow-up data collection: 1994).	Univariate distributions	- Desire to have future children<br>- FP methods used<br>- Intended/unintended pregnancies<br>- Intended/unintended births<br><br>Early approach aligned with **original definition** of unmet need.	**Prevalence:**<br>- **1991/92:** unmet need: 18%<br>- **1994:** unmet need: 17%	**Persistence of unmet need:**<br>- 72% women with unmet need for any FP initially had need satisfied.<br>- 43% with unmet need for modern FP initially had need satisfied.<br>- Only 17% women with met need, but 32% with unmet need had had an unintended pregnancy.<br>- Relative effectiveness of FP programs to reduce unintended births: 2.8-13.8%.
Machiyama and Cleland. 2013, Ghana	Determinants of non-use of contraception among women with unmet need for FP.	Cross-sectional household-based survey (DHS 2008) of 1,294 married or cohabitating women between the ages of 15-49.	Univariate distributions and multivariate regression analysis	- Woman's approval of FP<br>- Partner's approval of FP<br>- Woman's access to FP (physical/financial)<br>- Woman's knowledge of FP<br>- Woman's concerns of adverse health effects<br>- Frequency of sexual intercourse<br>- Currently breastfeeding<br>- Geographical region<br>- Woman's level of education<br>- Poverty level<br>- Time since last child birth<br>- Parity<br>- Woman's age<br>- Woman's religion (Christian/Muslim/Traditional)<br><br>Unmet need estimates based on **revised definition**.	**Prevalence:**<br>- Unmet need: 42%<br><br>**Association with unmet need:**<br>- Southern provinces: (+)<br>- Educational level woman: (-)<br>- Being Catholic: (-)<br><br>**Associations with access to FP:**<br>- All variables: n.s.s.<br><br>**Association with FP approval:**<br>- Being poor: (+)<br>- Last childbirth less 2 years: (+)	**Reasons for unmet need:**<br>- Lack of access: 45%<br>- Disapproval of FP: 43%<br>- Health concerns: 43%<br>- Disapproval of, but not access to FP reported as main reason

*n.s.s. = not statistically significant association, (+) = positive statistically significant association, (-) = negative statistically significant association

DHS = Democratic Health Survey; FP = family planning

Authors, year, location	Study objective	Study design	Analytical approach	Explanatory variables used	Statistical findings / associations with main outcome*	Other important findings
Mekonnen and Worku. 2011, Ethiopia	Prevalence and determinants of unmet need in Butajira District.	Cross-sectional household-based survey of 5,746 women between the ages of 15-49. (data collection: 2009).	Univariate distributions and multivariate regression analysis	- Residence (rural/urban)<br>- Woman's educational level<br>- Availability of food<br>- Experience of child death<br>- Partner's educational level<br>- Paid work status<br>- Couple discussing FP<br>- Partner's approval of FP<br>- Knowledge of FP<br><br>Unmet need estimates based on **revised definition**.	**Prevalence:**<br>- Unmet need: 52% (39% for spacing, 13% for limiting)<br>- FP use: 25%<br><br>**Association with unmet need (limiting / spacing / total):**<br>- Rural residence : n.s.s./(+)(+)<br><br>**Association with non-use:**<br>- Rural residence: (+)<br>- Education level woman: (-)<br>- Education level partner: (-)<br>- Child death: (-)<br>- Couple discussing FP: (-)<br>- Approval partner: (-)	**Knowledge and use of FP:**<br>- FP methods women have most knowledge of: hormonal implants (89%), oral hormones (98%), male condoms (82%)<br>- Most frequently ever-used FP methods: hormonal implants (98%), oral hormones (97%), male condoms (82%)
Ndaruhuye et al. 2009, Rwanda	Determinants of demand and unmet need for limiting.	Cross-sectional household-based survey (RDHS 2005) of 4,817 cohabitating women between the ages of 15-49.	Univariate distributions and multivariate regression analysis	- Woman's age<br>- Woman's level of education<br>- Woman's paid work status<br>- Woman's religion<br>- Woman's residence<br>- Geographical region<br>- Partner's level of education.<br>- Partner's paid work status<br>- Partner's approval of FP<br>- Poverty level<br>- Woman's knowledge on FP<br>- Exposure to FP information<br>- Woman's approval of FP<br>- Woman's perception on partner's approval of FP<br>- Couple discussing FP<br><br>Unmet need estimates based on **original definition**.	**Prevalence:**<br>- Unmet need 58% (16% for limiting)<br><br>**Association with limiting (demand / unmet need):**<br>- Education level woman: (-)/(-)<br>- Paid work status partner: (-)/n.s.s.<br>- Partner desires more children: n.s.s./(+)<br>- Exposure to FP info: (+)/(-)<br>- Knowledge on FP: (+)/(-)<br>- Approval FP woman: (+)/(-)<br>- Approval FP partner n.s.s./(-)<br>- Couple discussing FP: (+)/(-)<br>- All other variables: n.s.s.	**Other:**<br>- Association between women's educational level and demand for FP appears to be rather U-shaped.<br>- Association between women's educational level and unmet need for limiting appears to be rather linear.

*n.s.s. = not statistically significant association, (+) = positive statistically significant association, (-) = negative statistically significant association
DHS = Democratic Health Survey; FP = family planning

Authors, year, location	Study objective	Study design	Analytical approach	Explanatory variables used	Statistical findings / associations with main outcome*	Other important findings
Paudel et al. 2011, Nepal	Prevalence and determinants of unmet need for FP in Eastern Nepal.	Cross-sectional facility-based survey of 410 women of reproductive age attending child immunization services at a teaching hospital. (data collection: year not mentioned).	Univariate and multivariate distributions	- Woman's age<br>- Parity<br>- Family type (nuclear vs. joint)<br>- Woman's educational level<br><br>Unmet need estimates based on **original definition**.	**Prevalence:**<br>- Unmet need: 22% (15% for spacing, 8% for limiting)<br><br>**Associations with unmet need:**<br>- Age woman: (+)<br>- Parity (+)<br>- All other variables: n.s.s.	**Reasons for unmet need:**<br>- Adverse health effects: 31%<br>- Disapproval partner/family/ community: 14%<br>- Uncertainty child wish: 12%<br>- Inconvenience: 11%<br>- Lack of information: 4%<br><br>**Reasons for FP use:**<br>- Birth spacing: 80%<br>- Prevention unintended pregnancies: 75%<br>- Birth limiting: 63%<br>- Improving maternal/child health: 45%<br>- Reduction maternal death: 4%
Prateek and Saurabh**.** 2012, India	Prevalence and determinants of contraceptive use among women living in an urban slum community in Mumbai.	Cross-sectional facility-based survey of 180 women between the ages of 15-49 attending the general outpatient department of an urban health center. (data collection: year not mentioned).	Univariate and multivariate distributions	- Woman's knowledge of FP<br>- Woman's age<br>- Woman's religion (Hindu vs. Muslim)<br>- Woman's level of education<br>- Woman's socioeconomic status<br>- Woman's age at marriage<br>- Woman's age at first pregnancy<br>- Parity<br>- Partner's level of education<br><br>**KAP-gap Concept** of unmet need (based on married women, fecund/non-pregnant; limiting, only modern FP = *FP intention-use-gap*) closely aligned with **original definition** of unmet need.	**Prevalence:**<br>- Unmet need: 52%<br>- FP use: 32%<br><br>**<u>Association with non-FP use:</u>**<br>- Age woman (-)<br>- Being Muslim (+)<br>- Level of education woman: (-)<br>- Being poor: (+)<br>- Age at first pregnancy: (-)<br>- Level of education partner: (-)	**<u>Reasons for non-use:</u>**<br>- Lack of knowledge: 71%<br>- Desire for more children: 24%<br>- Concern of adverse health effects: 21%<br>- Opposition by family: 14%<br>- Not cohabitating with partner: 8%<br><br>**Reasons for use per method:**<br>- Condoms easily available: 36%<br>- IUP offers long-term protection: 28%<br>- IUD requires one time application: 24%<br>- Oral pill is comfortable: 12%<br>- Partner prefers oral pill: 10%<br>- Tubal ligation once family is completed: 10%<br>- Oral pill only method accepted by family: 3%

*n.s.s. = not statistically significant association, (+) = positive statistically significant association, (-) = negative statistically significant association
DHS = Democratic Health Survey; FP = family planning

Authors, year, location	Study objective	Study design	Analytical approach	Explanatory variables used	Statistical findings / associations with main outcome*	Other important findings
Sultan et al. 2010, Egypt	Prevalence and determinants of unmet need for FP in Eastern Cairo.	Cross sectional household-based survey of 2,340 cohabitating married women between the ages of 15-49. (data collection: 2009).	Univariate and multivariate distributions	- Woman's age<br>- Woman's age at marriage<br>- Number of children<br>- Number of male children<br>- Number of female children<br>- Age of last child<br>- Number of abortions<br>- Religion (Muslim vs. Christian)<br>- Woman's paid work status<br>- Partner's paid work status<br>- Woman's level of education<br>- Partner's level of education<br><br>Unmet need estimates based on **revised definition**.	**Prevalence:**<br>- Unmet need: 7% (4% spacing, 3% for limiting)<br><br>**Association with unmet need:**<br>- Number of children: (+)<br>- Number of girls: (+)<br>- Number of abortions: (+)<br>- All other variables: n.s.s.	**Reasons for FP non-use:**<br>- Concerns adverse health effects: 54%<br>- Perception of low risk of getting pregnant: 18%<br>- Breastfeeding: 15%<br>- Contraindication due to health problems: 8%<br>- Opposition to FP: 5%<br><br>**Reasons for FP discontinuation:**<br>- Menstrual side effects: 33%<br>- Non-menst. side effects: 19%<br>- Method failure: 14%<br>- Desire for child: 12%<br>- Other health problems: 9%
Umbeli et al. 2005, Sudan	Prevalence and determinants of unmet need for FP in Dar Assalam/Khartoum State.	Cross-sectional facility-based survey of 530 married women between the ages of 15-49 visiting the local health center. (data collection: 2001).	Univariate and multivariate distributions	- Knowledge of FP<br>- Number of children<br>- Woman's level of education<br><br>Unmet need estimates based on **original definition**.	**Prevalence:**<br>- Unmet need: 31%<br><br>**Association with FP use:**<br>- Desire for more children: (-)<br>- Educational level woman: (+)	**Source of FP knowledge:**<br>- Relatives: 49%<br>- FP outreach program: 24%<br>- Mass media: 11%<br>- Midwife: 10%<br>- School: 4%<br>- Doctor: 3%
Wablembo et al. 2011 Uganda	Effect of spousal discussion on unmet need for FP.	Cross sectional household-based survey (UDHS 2006) of 8,531 married or cohabitating women between the ages of 15-49.	Univariate distributions and multivariate regression analysis	- Woman's age<br>- Woman's level of education<br>- Partner's level of education<br>- Poverty level<br>- Religion (Muslim vs. Christian)<br>- Residence (urban vs. rural)<br>- Number of children<br>- Couple discussing FP<br>- Partner's desire for children<br>- Woman's desire for children<br><br>Unmet need estimates based on **original definition**.	**Prevalence:**<br>- Unmet need: 41%<br><br>**Association with unmet need:**<br>- Age woman: (-)<br>- Education level woman: (-)<br>- Education level partner: (-)<br>- Rural residence: (+)<br>- Being Muslim: (-)<br>- Being poor: (+)<br>- Number of children: (+)<br>- Number couple discussion: (-)<br>- Desire for additional child woman: (-)	

*n.s.s. = not statistically significant association, (+) = positive statistically significant association, (-) = negative statistically significant association

DHS = Democratic Health Survey; FP = family planning

Authors, year, location	Study objective	Study design	Analytical approach	Explanatory variables used	Statistical findings / associations with main outcome*	Other important findings
Wolde-micael and Beaujot. 2011, Eritrea	Prevalence and determinants of unmet need for FP.	Cross- sectional household-based survey data (EDHS 1995 and 2002) of 8,531 married women between the ages of 15-49.	Univariate distributions and multivariate regression analysis	- Woman's age<br>- Number of living children<br>- Woman's level of autonomy<br>- Couple discussion on FP<br>- Poverty level<br>- Woman's level of education<br>- Residence (urban vs. rural)<br>- Geographical region<br>- Knowledge of and access to FP<br><br>Unmet need estimates based on **original definition**.	**Prevalence:**<br>- 1995: Unmet need: 28% (21% for spacing, 6% for limiting)<br>- 2002: Unmet need: 27% (21% for spacing, 6% for limiting)<br>- 1995: FP use: 8% (6% for spacing, 2% for limiting)<br>- 2002: FP use: 8% (5% for spacing, 3% for limiting)<br><br>**Association with unmet need (spacing / limiting / total) (2002 only):**<br>- Age woman: (-)/n.s.s./(-)<br>- Number children: (+)/(+)/(+)<br>- Autonomy woman: (-)/(-)/(-)<br>- Couple discussion: (+)/(+)/(+)<br>- Being poor: (+)/n.s.s./n.s.s.<br>- Level of education woman: n.s.s./(+)/(+)<br>- Rural residence: (-)/n.s.s./(-)<br>- Red Sea region: n.s.s./(-)/n.s.s.<br>- Knowledge/access: (-)/(-)/(-)	**Reasons for non-use (2002 only):**<br>- No knowledge on FP: 19%<br>- No knowledge on access: 17%<br>- No/infrequent intercourse: 16%<br>- Disapproval woman: 15%<br>- Postpartum/breastfeeding: 7%<br>- Religious belief: 6%<br>- Infecund: 5%<br>- Concern of adverse health effects: 5%<br>- Menopausal/hysterectomy: 5%<br>- Disapproval partner: 3%<br>- No access to FP: 2%
Yadav et al. 2009, India	Level of agreement on unmet need for FP among couples.	Cross sectional household-based survey of 200 married women between the ages of 15-44 and their partners in Dayalpur village/Haryana. (data collection: 2003-05).	Univariate and multivariate descriptive analysis, kappa statistics	- Fertility preference woman Fertility preference partner<br><br>Unmet need estimates based on **revised definition**.	**Prevalence:**<br>- Unmet need women: 18% (6% for spacing, 12% for limiting)<br>- Unmet need partners: 11% (4% for spacing, 8% for limiting)<br><br>**Level of agreement on unmet need:**<br>- Concordance of 94% among couples regarding unmet need (83% no unmet need, 7% unmet need)<br>- Discordance of 7% (women reporting unmet need, partner reporting no unmet need)	-

*n.s.s. = not statistically significant association, (+) = positive statistically significant association, (-) = negative statistically significant association
DHS = Democratic Health Survey; FP = family planning

Charting Form 2: Mapping of reviewed multi-country quantitative studies on determinants of unmet need for family planning and/or non-contraceptive use in low- and middle-income countries..

Authors, year, location	Study objective	Study design	Analytical approach	Explanatory variables used	Statistical findings / associations with main outcome*	Other important findings
Bongaarts and Bruce. 1995, LMIC	Causes of unmet need for FP and contraceptive use; role of FP programs.	- Review of data from 27 DHS collected between 1986-90 (estimates and determinants of FP use and need).<br>- Review of DHS data, program evaluations, and ethnographic data between 1987-94 (FP program implications).	Univariate and multivariate distributions	- Level of a country's development<br>- Distance to nearest FP service<br>- Knowledge of FP<br><br>Early approach aligned with **original definition** of unmet need.	**Prevalence:**<br>- **Unmet need**: SSA (23%), LA (19%), NAME (19%), Asia (14%)<br>- **FP use:** LA (48%), Asia (47%), NAME (41%), SSA (16%)<br><br>**Associations with unmet need and FP use:**<br>- The higher the HDI, the lower the total unmet need and unmet need for spacing.<br>- Unmet need for limiting lower when very high or very low HDI.<br>- The higher the HDI, the higher FP use.<br>- No statistically significant association between unmet need and distance to FP service.<br>- The higher the knowledge of FP, the higher the unmet need	**Reasons for unmet need:**<br>- Lack of knowledge: 25%<br>- Concern adverse effects: 20%<br>- Disapproval partner: 9%<br>- Infrequent intercourse: 6%<br>- Religious beliefs: 4%<br>- Lack of physical access: 4%<br>- Disapproval woman: 4%<br>- Lack of financial access: 3%<br>- Fatalism: 3%<br>- Inconvenience using FP: 2%<br>- Disapproval fam./comm.: 1%<br><br>**Other:**<br>- Only few fertility/FP studies include males/sexual partners/<br>- DHS: unmet need not directly explored, but inferred from discrepancy stated FP intentions vs.current FP use.<br>- Uncoupled and unmarried women often neglected in unmet need estimations.<br>- 34-73% couples never discussed FP<br>- FP use higher among couples where partner approves FP.<br>- Determinants quality FP services: service availability, no undue personal/ psycho-logical costs, limited travel time, limited monetary costs<br>- Determinants of low knowledge: gender roles, low literacy, no geographical movement, low service outreach.

DHS = Democratic Health Survey; FP = family planning; HDI = Human Development Index; LA = Latin America; LMIC = low- and middle-income countries; NAME = North Africa/Middle East; SSA = Sub-Saharan Africa

Authors, year, location	Study objective	Study design	Analytical approach	Explanatory variables used	Statistical findings / associations with main outcome*	Other important findings
Jain et al. 2013, LMIC	Determinants of modern FP use, any FP use, and unmet need for FP after FP discontinuation at country level.	Review of DHS data collected between 2005-10 of married women in 34 countries (determinants of unmet need).<br>Review of FPES 2009 data (availability modern FP).	Univariate distributions and multivariate regression analysis	- Number of available FP methods in country<br>- Type/mix of available FP in country<br>- Access to available FP methods<br>- Country's level of development<br>- Global region<br><br>Unmet need estimates based on **revised definition**.	**Prevalence:**<br>- **Unmet need**: 27% (31% for SSA, 25% for other LMIC)<br>- **FP use:** 36% (18% for SSA, 47% for LMIC)<br><br>**Associations with unmet need:**<br>- Access to FP: (-)<br>- Availability long-acting/ permanent FP methods: (-)<br>- Living in SSA: (+)<br>- HDI: n.s.s.	**Other:**<br>- In settings with higher FP use, increase related to higher proportion of long-acting/ permanent FP method users.<br>- Effect of access to and FP mix on FP discontinuation transmitted through FP use.
Nortman. 1982, Bangladesh, Colombia, Costa Rica, Korea, Mexico, Thailand	Determinants of demand for FP in six low- and middle-income countries.	Cross-sectional household-based survey (CPS 1978/-79) of married women of reproductive age.	Univariate and multivariate distributions	- Woman's age<br>- Experience of child death<br>- Couple's demand for FP<br>- Couple-years of FP need<br><br>Early approach aligned with **original definition** of unmet need.	**Prevalence unmet need (spacing / limiting / total):**<br>- Bangladesh: 37%/30%/67%<br>- Colombia: 11%/28%/39%<br>- Costa Rica: 12%/10%/22%<br>- Korea: 7%/22%/29%<br>- Mexico: 20%/24%/44%<br>- Thailand: 13%/21%/34%	**Other:**<br>- Total unmet need higher in women <30 years of age<br>- Unmet need for spacing higher in women <30 years of age.<br>- Unmet need for limiting higher in women >30 years of age.<br>- Unmet need lower in women who experienced child death.
Pearson and Becker. 2014, Benin, Burkina Faso, Mali	Prevalence of unmet need for FP among married couples.	Review of DHS data (Benin 2006, Burkina Faso 2003, Mali 2001) of 13,973 married couples.	Univariate and multivariate distributions	- Fertility preference woman<br>- Fertility preference partner<br><br>Unmet need estimates based on **revised definition**.	**Prevalence unmet need woman (spacing / limiting / total):**<br>- Benin: 14%/7%/21%<br>- Burkina Faso: 20%/6%/27%<br>- Mali: 22%/8%/30%<br><br>**Prevalence unmet need partner (spacing / limiting / total):**<br>- Benin: 12%/5%/17%<br>- Burkina Faso: 17%/3%/20%<br>- Mali: 18%/2%/20%<br><br>**Prevalence unmet need couples (Benin / Burkina Faso / Mali):**<br>- Woman only: 9%/13%/16%<br>- Partner only: 4%/6%/6%<br>- Both: 11%/13%/14%	**Unmet need ratios couples (Benin / Burkina Faso / Mali):**<br>- Ratio woman only / all women: 0.45 / 0.50 / 0.52<br>- Ratio partner only / all women: 0.27 / 0.23 / 0.21<br>- Ratio both/ all women: 0.55 / 0.50 / 0.48<br>- About half of women's unmet need in couples with discordant fertility preferences.<br>- Considering partners' unmet need identifies additional 21-27% couples with at least one partner having unmet need.

CPS = Contraceptive Prevalence Survey; DHS = Democratic Health Survey; FP = family planning; FPES = Family Planning Program Effort Survey; HDI = Human Development Index; LMIC = low- and middle-income countries; SSA = Sub-Saharan Africa

Authors, year, location	Study objective	Study design	Analytical approach	Explanatory variables used	Statistical findings / associations with main outcome*	Other important findings
Ross and Winfrey. 2001, LMIC	Prevalence of unmet need for FP, FP use, and intention to use during extended postpartum period.	Review of data of 27 DHS of 27 countries conducted between 1993-96 of women with last delivery in past 12 months.	Univariate and multivariate distributions	- Woman's age<br>- Global region<br><br>Early approach aligned with **original definition** of unmet need.	**Prevalence unmet need (spacing / limiting / total)**:<br>- SSA (55%/19%/74%)<br>- LAC (21%/33%/54%)<br>- NAME (23%/29%/53%)<br>- Asia (33%/29%/62%)<br><br>**Prevalence FP use (spacing / limiting / traditional / total)**:<br>- SSA (6%/3%/9%/18%)<br>- LAC (11%/19%/12%/42%)<br>- NAME (10%/18%/16%/44%)<br>- Asia (13%/12%/7%/32%)	**Intention to use FP beyond 12 months after last delivery:**<br>- SSA (23%); LAC (78%); NAME (11%); Asia (11%)<br><br>**Intention to use FP among women with unmet need:**<br>- SSA (58%); LAC (13%); NAME (76%); Asia (67%)<br><br>**Predictability actual FP use based on intention to use:**<br>- Where stated intention to use FP is high within first 3 months postpartum, actual FP use rises substantially within 12-months postpartum period.<br>- For each 1% increase intention to use, there is about 1% rise in FP adoption.
Ross and Winfrey. 2002, LMIC	Prevalence of unmet need for FP in developing world.	Review of data of 55 DHS of 115 countries conducted between 1990-99 of married women.	Univariate and multivariate distributions	- Woman's age<br>- Global region<br><br>Early approach aligned with **original definition** of unmet need.	**Prevalence unmet need (spacing / limiting / total)**:<br>- SSA (16%/9%/24%)<br>- LAC (6%/8%/14%)<br>- NAME (6%/9%/16%)<br>- Asia (14%/14%/28%)<br><br>**Prevalence unmet need LMIC:**<br>- 1996: 19%<br>- 2000: 17%	**Other:**<br>- Women age 15-24 make 33% of all married women with unmet need in LMIC.<br>- Estimates of unmet need would be considerably larger under assumption that users of traditional methods have unmet need for modern methods.

DHS = Democratic Health Survey; FP = family planning; LAC = Latin America and Caribbean; LMIC = low- and middle-income countries; NAME = North Africa and Middle East; SSA = Sub-Saharan Africa

Charting Form 3: Mapping of reviewed review studies on determinants for unmet need for family planning and/or non-contraceptive use in low- and middle-income countries.

Authors, year, location	Study objective	Study design	Analytical approach	Key findings
Casterline and Sinding, 2000, LMICs	Utility of unmet need for FP concept reproductive health planning and FP programming purposes.	Review of multi-country DHS data, qualitative, and mixed studies published between 1995-2000.	Mixed methods review	**Unmet need as a concept:**<br>- Unmet need often not directly based on respondents' reported information, but rather on inferences made by researcher: 1) those women stating desire to space/limit childbearing; 2) those in 1) who would like to take actions to avoid births in form of FP rather than induced abortion or any other actions.<br>- Unmet need likely underreported as current empirical practice excludes (temporarily) infecund women from being included into analysis.<br>- Direct evidence for existing unmet need as concept provided by high incidence of reported unintended pregnancies (20-25% of births estimated in developing world to be unwanted).<br>- Many women and men feel frustrated by their inability to adopt behaviors effectively preventing unintended pregnancies.<br>- As with unmarried women, there has been limited empirical research on men's unmet need for FP.<br><br>**Relationship between unmet need and met need for FP:**<br>- Reduction in fertility (i.e. increase in FP use) among those women at risk for unwanted births represents a disproportionate contribution to overall reported fertility decline.<br>- Substantial increases in prevalence of FP use (i.e. substantial decline in fertility) can be achieved in absence of changes in demand for children through meeting the need for FP.<br><br>**Relationship between unmet need and demand for FP**:<br>- Category "unmet need" often composed of women who vary considerably in their demand for FP.<br>- Many women with unmet need remain unlikely to adopt FP use any time soon – not primarily due to lack of access, but because of extreme reluctance to FP use due to negative perception of not conceiving (i.e. social, cultural, and health concerns).<br>- Only fraction of unmet need represents with a latent demand for FP susceptible to conversion into use.<br>- Only once FP practice is cost-free (including cultural, social, health, financial, and time costs) all women and men with unmet need could be regarded as having a latent demand for FP.<br>- Stronger correspondence between "intention to use" (not "unmet need for FP") and subsequent FP use.<br><br>**Redefining "unmet need":**<br>- Unmet need should include qualitative as well as quantitative dimensions.<br>- FP users may still have FP needs; high FP prevalence can coexist with significant unmet need for FP.<br><br>**Reasons for unmet need:**<br>- Lack of access<br>- Lack of knowledge<br>- Social disapproval<br>- Concerns of adverse health effects

DHS = Democratic Health Survey; FP = family planning

Authors, year, location	Study objective	Study design	Analytical approach	Key findings
Shaikh et al, 2010, Pakistan	Reasons of unmet need for FP	- Review of PDHS data from2006-2007 data<br>- Review of published and grey literature	Scoping Review	**Reasons for non FP use:**<br>- **User Level:** social relations (e.g. partner's opposition, desire for more children), low female literacy, method-related misconception or fear of FP); lack of culturally-sensitive IEC/behavior change programs<br>- **Family Level:** opposition from family members, lack of support from mother-in-law, dependency on family norms, lack of decision-making autonomy of women (including health and emergency situations); lack of engagement of FP programs with older women at community level.<br>- **Community Level:** persistent myths about modern FP, religious opposition/misinterpretation; lack of adequate social marketing, lack of appropriate health-seeking behavior communication, lack of grassroots program making FP acceptable as a social norm<br>- **Health Service Level:** limited access, poor service quality, ineffective promotion of modern FP, weak public sector outlets (especially in rural/peri-urban areas)<br>- **Other Levels:** social marketing through radio/TV often not sufficiently open and direct, outreach hampered by poor traffic infrastructure, little alignment between youth/women development programs and health/population strategies; lack of use of electronic media for social marketing<br>- **State Level:** poor integration of FP services in primary health care, little cross-sectional strategies (e.g. promotion of girls' education), poor investment and evaluation of social marketing campaigns/FP programs, donor funding oriented toward vertical programs, hampering government policies
Westoff and Bankole, 2000, LMICs	Demand for family limitation in developing countries	Review of data of 108 DHS, WFS, and CPS reports from 41 countries from mid-1970s to late 1990s.	Systematic Review	**Prevalence:**<br>- **Unmet need for limiting (range; lowest/year; highest/year):** Sub-Saharan Africa 1977-98 (4-26%; Benin/1981, Cote d'Ivoire/1980; Kenya 1989), Asia & North Africa 1975-98 (9-31%, India/1992; Pakistan/1995), Latin America & Caribbean 1975-98 (10-39%; Colombia/1994; Bolivia/1989);<br>- **Unmet need for limiting** increases in Sub-Saharan Africa, at same time declines in the other regions<br>- **Total potential demand for limiting** (i.e. sum of FP use and unmet need for limiting): increased in all regions (still raising in Sub-Saharan Africa, beginning to level off in other regions); highest in Latin America, lowest in Sub-Saharan Africa; relative percentage-point increase in demand largest in Sub-Saharan Africa (only region where both current use and unmet need are increasing simultaneously).<br>- **Unmet need** is a moving target, rising in early stages of the transition as interest in family limitation grows, declining in the later stages when FP use is adopted.

CPS: Contraceptive Prevalence Surveys: DHS: Democratic Health Survey; FP = family planning; WFS: World Fertility Survey

Charting Form 4: Mapping of reviewed qualitative studies on determinants for unmet need of family planning and non-use of contraceptives in low- and middle-income countries

Authors, year, location	Study objective	Study design	Analytical approach	Main themes, thematic relationships
Bawah et al. 1999, Ghana	Acceptability of FP program by community and impact of FP on gender relations in Northern Ghana.	Community-based before-and-after study including a total of 36 FGDs with 10 session at baseline in 1994 (2 sessions each for older men and older women ages 45-65, younger men and younger women ages 20-29, opinion leaders of the community), 10 sessions after pilot implementation in 1995 (same as in 1994), and 16 sessions after program scale-up in 1996 (only gender and age groups, no opinion leaders).	Content analysis of transcripts after translation into English.	**WOMEN'S INTEREST IN FP USE:**<br>- **Role of women within marriage:** obligation to fulfill partner's sexual desires in order to avoid spousal discord, threat of violence; women are responsible for childcare.<br>- **FP need**: almost entirely a desire to space rather than to limit; women perceive modern FP valuable for meeting the need for spacing. FP need stronger among women compared to men; women more willing to talk about FP use compared to men.<br>- **Intention to use FP:** FP allows women pursuing own reproductive strategies. Women perceive FP as their right, given women's sole responsibility for child-care under conditions of extreme deprivation.<br>**WOMEN'S FEAR OF OPPOSITION/REPRISAL TO FP USE:**<br>- **Spousal disapproval and gender roles:** women have little decision-making autonomy; women proceeding to use FP in face of husbands' opposition do violate gender norms; tensions arise when a) husbands disagree/distrust their wives' FP intentions, or b) women are too afraid to raise FP discussions with their husbands.<br>- **Spousal disapproval and domestic violence:** domestic violence, including wife-beating, considered a legitimate form of spousal interaction by many women; women using FP methods without husband's knowledge causes men's disapproval and result in domestic violence.<br>- **Spousal disapproval and polygamist marriage:** husbands' disapproval of FP use can lead to withholding affection, withholding sex, divorce, or preference for another wife in polygamous marriages; non-use of FP might appear preferable to women in polygamous context where a woman must compete with other wives.<br>- **Spousal disapproval and access to FP:** procuring money for FP from one's husband can provoke conflict; even if actual costs for FP are small, emotional costs of pursuing FP negotiations can be great.<br>- **Family/ancestral disapproval:** nowadays family members have less influence on couple's FP decisions; some husbands understand the need for FP, their mothers and fathers might still disagree; persisting traditional beliefs among women that ancestors are against FP practices, which leads to ancestral disapproval with withdrawal of spiritual blessings or even a woman's death when practicing FP.<br>**WOMEN'S COPING STRATEGIES USING FP**:<br>- **Autonomy and social respect:** women adopt complicated/subtle reproductive/sexual strategies to achieve some degree of sexual autonomy while still maintaining their social position in family and community.<br>- **Examples for coping strategies:** to give husband impression FP practice is his own decision; to create additional sources of income in order to purchase FP independently of husband's approval; to resist unwanted sexual advances by discrediting husband within community; to secretly use of FP.<br>**MEN'S FEAR USING FP:**<br>- **Social norms of marriage:** wives are acquired through bride-wealth payments (sheep, cattle); women are therefore considered husbands'/husband's family's property; married women obligated to bear children in return; women taking control of reproduction suggests their wish to not meet marriage

				obligations, posing threat to strong patriarchal traditions.<br>- **Male perception of FP:** FP programs disturb existing social order by providing women living in traditional and gender-stratified societies with technology facilitating control of reproduction,.<br>- **Reproductive obligations:** steady supply of children considered essential by society that depends on strong male lineages; while women's reproductive focus is primarily on assuring health of their offspring (e.g. through spacing), men's reproductive focus emphasizes the security of having many children (to overcome threats posed by child mortality/infertility).<br>- **Fear of infidelity/promiscuity:** women practicing FP are perceived as unfaithful to their husbands or inclined to abandon their families; from male perspective potential loss of spouse creates ridicule of man's status/honor/pride and represents major economic threat given initial bride-wealth investment.<br>- **Fear of losing control:** men are sole decision-makers; FP decisions require therefore husband's consent; women do agree that husband's authority is critical; women who act independently on FP decisions and encounter problems due to FP use are thus to be blamed for damaging themselves and the family.<br>**CONSIDERATIONS FOR FP PROGRAMMING:**<br>- **Active involvement** of local leaders (chiefs, village committees, community assembly members) and women groups in community health and FP themes.<br>- **Outreach to men** by field workers using village-level male associations (farming, drumming), home visits.<br>- **Support for women** by protecting women's interests and safety; this requires sustained attention, worker training, and frequent meetings with service-delivery staff.

FGD = focus group discussion; FP = family planning

Authors, year, location	Study objective	Study design	Analytical approach	Main themes, thematic relationships
Hall et al. 2008, India	Barriers to FP use among rural women in Western India.	Community-based cross-sectional study including a total of 6 FGD with each 8-14 ever-married women between the ages of 19-50 and 15 semi-structured in-depth interviews with currently married women between the ages of 25-53.	Content analysis of transcripts after translation into English.	**FP USE:**<br>- **FP methods used:** a) FGD: periodic abstinence and female sterilization most commonly practiced methods; b) Interviews: attitudes towards modern FP methods differ depending on educational/ socioeconomic backgrounds; copper IUD is most commonly used reversible FP method; abortion considered back-up when a woman conceived shortly after giving birth or abstinence method failed.<br>- **Reasons for FP use:** a) Spacing: allows physical recovery from labour before next pregnancy; prevents challenges of household duties due to multiple infants; allows offering full course of breastfeeding to each infant; b) Limiting: allows smaller family size; allows adequate education for each child, allows improving social family's status.<br>- **FP and social norms:** concept of 'self control' (i.e. abstinence and/or withdrawal) most frequently practiced and considered an admirable personal virtue; having three or more children considered irresponsible, however, preference for male offspring makes women with female offspring to bear additional children; women reluctant to adopt long-term FP methods as they cannot reversed easily when husbands is away, which implies female infidelity; abortion considered dangerous, shameful (result of sexual immoderation), and needs to be concealed.<br>**SOCIAL RISKS:**<br>- **Family pressure:** large family size considered an ideal by extended family; older relatives pressure couples to continue bearing children and forbid use of FP; Hindu culture includes a number of menstrual taboos – close proximity of extended family members makes secret FP use difficult as menstruation cycles of a women is indirectly observed by household members.<br>**ECONOMIC RISKS:**<br>- **Vasectomy vs. tubal ligation:** female sterilization more prevalent than male sterilization despite easy availability, no cost, and much higher incentive payment for vasectomy; general believe vasectomy to permanently disable men from doing hard labour, thus removing primary source of household income.<br>**SIDE EFFECTS:**<br>- **IUD:** believed to damage uterus and health, as IUD causes heavier, longer, more frequent menses; IUD users face exclusion from domestic/religious activities due to change in menses (menstrual taboo).<br>- **Oral contraception:** hardly used for FP, instead frequently used to circumvent menstrual taboo by taking OCP over only few days to delay menstruation; OCP abuse and overdose without physician guidance, leading to excess side-effects (abdominal pain, nausea, vomiting, vertigo, vaginal infections).<br>- **Vasectomy:** potential vasectomy failure as strong disincentive for women as this casts doubt on fidelity of wife; most women consider female sterilization better option regardless of more invasive nature.<br>**INFORMATION AND KNOWLEDGE ON FP:**<br>- **Source of information:** primarily through female relatives and friends; educated women through school; FP officials never visited or treat women rudely.<br>- **Information content:** only information about sterilization, hardly about other FP methods.<br>- **Knowledge of FP:** periodic abstinence most frequently mentioned, but incorrectly practiced.

FGD = focus group discussion; FP = family planning

Authors, year, location	Study objective	Study design	Analytical approach	Main themes, thematic relationships
Kabagenyi et al, 2014, Uganda	Reasons and barriers for men's limited reproductive health involvement in rural Uganda.	Community-based cross-sectional study including a total of 18 FGDs (8 with a total of 70 married and unmarried men between the ages of 15-54; 10 with a total of 84 married and unmarried women between the ages of 15-49), and 8 key informant interviews (district health officers, village health team members, council leaders, representatives of local women and men's groups).	Content analysis of transcripts after translation into English.	**DISSATISFACTION WITH FEMALE FP METHODS:**<br>- **Male perception of female FP:** women are perceived to carry the physical burden of FP side effects, men however consider themselves indirectly affected by FP side effects (women's reduced sexual pleasure, increased risk of female infertility/illness, irregular/prolonged bleeding, vaginal dryness, decreased libido); concerns of FP induced delayed return to fertility, permanent loss of fertility, congenital abnormalities; FP side effects cause adverse economic effects on household as result of women's reduced ability to endure physical demands of agricultural labor; additional medical care costs to treat women's discomfort due to FP use.<br>- **Male coping strategies:** especially increased bleeding is considered as limiting the opportunities for men to have sex with their partner, and serves as motivation for men to develop extramarital sexual relations; requests by men for spouses to discontinue FP use altogether.<br>**DISSATISFACTION WITH MALE FP METHODS:**<br>- **Unmet need for male FP:** main incentive for FP is economic burden of raising large families; only limited access to male-led method (except for condom, vasectomy) which results in additional disapproval of FP by men; vasectomy perceived as losing one's masculinity, thus inacceptable FP option for men; relative strong male interest and preferences for male version of birth control pills.<br>- **Female perception of male FP:** older men consider condoms designed for unmarried/younger men, not tailored to older sexually active men; use of condoms associated with distrust among couples.<br>**FP AS WOMAN'S DOMAIN:**<br>- **Gender roles:** role of childbearing, child-rearing, fertility and FP assigned to women; generating family income assigned to men; use of men's limited time and mental preoccupation to discuss FP considered burdensome and distractive.<br>- **Inappropriate programming of FP:** traditionally FP services and campaigns target only women, thus further defining FP as a woman's domain; lack knowledge on how men could get involved in FP even if they wanted to; especially birth spacing produces negative attitudes towards FP among men.<br>**PREFERENCE FOR LARGE FAMILY SIZE:**<br>- **Patrilineal tradition:** high value of many children (wealth, financial security) and large family size; FP undermines husband's fertility desires.<br>- **Religious/traditional beliefs:** having as many children as possible considered divine directive for couples of reproductive age; FP has no moral legitimacy in this context.<br>**FP AND SEXUAL PROMISCUITY:**<br>- **Men's fear of female promiscuity:** FP induced potential infidelity of woman disengages supportive male roles in FP; FP use enhances women's physical attractiveness to other men (delayed or prevented childbearing), thus require husband's constant effort to defy other men's sexual interests in own spouse.<br>- **Stigma of FP use:** beliefs that FP use reflects women's intentions to avoid pregnancy within extramarital sexual relationships or in context of female commercial sex exchange; FP not considered socially acceptable to use for faithful, married women.

FGD = focus group discussion; FP = family planning

Authors, year, location	Study objective	Study design	Analytical approach	Main themes, thematic relationships
Kaida et al. 2004, Uganda	Male participation in FP.<br>.	Community- and facility-based cross-sectional study including 4 FGD (1 with married men currently using FP, 1 with married men currently not using FP, 1 with male FP health staff, 1 with female FP health staff) and 7 key informant interviews (DHMT members, private FP providers, town council members, representatives of Family Planning Association of Uganda (FPAU)).	Content analysis of transcripts after translation into English.	LACK OF INFORMATION/MISCONCEPTIONS:<br>- **Knowledge of FP:** lower among men compared to women; most men could name at least one FP method, only few understood how this particular method actually worked<br>- **Source of FP knowledge:** men receive FP information mainly from the radio.<br>- **Fear of FP use:** rumors about oral contraceptives to be inefficient in preventing pregnancies, to create permanent infertility or cause congenital malformations; rumors about condoms to spread HIV.<br>**FP SIDE-EFFECTS:**<br>- **Male concerns regarding FP side-effects:** general sickness, menstrual disturbance, weight gain/loss, nausea, weakness, infertility, malformation of newborns; concerns about side-effects more pronounced among men with limited access to health care facilities/FP information.<br>**FP SERVICES/SUPPLIES:**<br>- **FP availability:** unavailability of FP important barrier for men using FP; supply of FP devices generally unreliable; only very limited range of FP methods available due to national shortages.<br>- **FP access:** long distance to nearest FP outlet as barrier receiving FP services; relatively high cost/low availability of transportation/time to visit clinic; men perceive their accessibility to FP service generally lower as women.<br>**FP PERSONNEL:**<br>- **Skepticism/distrust:** intentions and motivation of FP personnel/programs encouraging individuals to use FP unclear to users and non-users; providers seem more concerned with own financial/personal gains than needs of community; concerns that FP providers withhold important information about FP associated health risks;<br>- **Perceived quality of FP services:** providers sometimes rude, dismiss/disregard clients' fears about side-effects; although local language is used to communicate with clients, most FP supplies are from abroad with instructions for use not translated into local languages.<br>- **Male perception of FP services:** men do not feel welcome at FP centers; fear discussing sensitive and private issues with female provider.<br>- **Couple counselling:** counselling of couples by FP providers is rare; providers prefer to counsel men and women separately; female FP providers more comfortable counselling women than men; different information about FP given to men and women contributes to more mistrust between couples.<br>COUPLE COMMUNICATION/TRUST:<br>- **Lack of communication between couples:** attributed to perception that FP use is associated with promiscuity/infidelity; men report to be suspicious if wives make decision to use FP on their own.<br>- **Secret use of FP:** high demand of injectable FP due to secret use of FP by women without husband's knowledge/approval.<br>**CULTURAL AND RELIGIOUS FACTORS:**<br>- **Number of children/family size:** men express desire for many children, which enhances prestige in community, ensures labour and social security; counteracts high rate of child mortality.<br>- **Patriarchal/patrilineal/polygamist society:** preference for sons, as boys inherit father's clan and thus increase clan's power, status, longevity; competition between wives for husband's love often played out with number of children/sons borne by each woman.<br>- **Religion** widely considered as barrier to contraceptive use, Anglican Church more accepting of modern methods compared to Catholic and Muslim denominations.

				**MOTIVATORS ENCOURAGING MALE INVOLVEMENT:**<br>- **FP services:** improve FP information, education, sensitization specifically directed at men; providing FP information during community seminars conducted by a well-respected local individual; making FP information available where men are (e.g. workplaces, bars, community areas where men tend to congregate); ensure some FP providers to be men; ensure male FP choices to be available.<br>- **Couples communication:** encourage communication between husbands and wives, between providers and couples to serve as motivator to stimulate male involvement in FP; counselling couples together may alleviate some distrust associated with a woman approaching her husband on practicing FP; couples counseling however should be considered secondary to ensure a woman's FP needs are met even if husband is against FP use.
Mosha et al. 2013, Tanzania	FP decisions, perceptions and gender dynamics among couples in Mwanza region.	Community-based cross-sectional study including 12 FGD (6 with 50 married or cohabiting females aged 18-49, 6 with 48 married or cohabiting males aged 18-49) and 6 key informant interviews (3 females and 3 males each married or cohabiting between the ages of 18-49).	Content analysis of transcripts after translation into English.	**RISKS/COSTS OF FP:**<br>- **Male concerns:** FP use associated with marital infidelity as FP methods allow women to have extra-marital affairs without being discovered since they would not be able to conceive; women can use FP only after approval by partner<br>- **Female concerns:** FP as a sign of a woman's faithlessness; general lack of knowledge of FP.<br>- **Male perception of side effects:** headaches, bleeding, weight gain/loss, nausea, dizziness, stomach-ache; infertility, cancer, birth deformities; belief that long-acting implants could travel throughout body, get lost, cause harm to user.<br>- **Female perception of side effects:** cancer, over-bleeding, uterine tumors, infertility<br>- **Financial implication of side effects:** male disapproval of wives using FP because of financial repercussions from treating wives if they experience side effects; female dependency on husbands for medical treatment in case of side effects.<br>**GENDER RELATIONS:**<br>- **Female interest in FP:** FP methods help to plan/space children and improve general health situation.<br>- **Secrecy in FP use:** women's role is child-rearing; some women use FP without their husbands' consent in order to protect their health and the plight of their children<br>- **Couple communication:** most couples nowadays discuss FP use because of economic hardship as results of large family size; compared to urban areas, in rural areas occur little communication among couples on FP use and desired number of children; discussing FP among couples considered irrelevant due to traditional (female role in FP) and religious reasons; women find it difficult to engineer discussions as men are main decision-makers.<br>**MALE INVOLVEMENT IN FP:**<br>- **Patriarchal norms:** men are heads of households and decision-makers in all issues including number of children and FP; men expected to initiate discussions on FP and number of the children a couple wants to have; women considered implementers of what has been decided by men without questioning men's decisions.<br>**URBAN/RURAL DIFFERENCES:**<br>- **Rural norms:** men express preference for large families; FP considered contrary to these preferences; preference of many children as they serve as labour force in farming activities; having as many children as possible facilitated by extended family support system; relatives influence number of children a couple might have; especially mother-in-laws put pressure on sons/daughter-in-laws to have more children than initially planned.<br>- **Urban norms:** value of children in terms of costs involved in raising children (school, medical services, social amenities); having more children would mean incurring more costs.

FGD = focus group discussion; FP = family planning

Authors, year, location	Study objective	Study design	Analytical approach	Main themes, thematic relationships
Plummer et al. 2006, Tanzania	Knowledge, attitudes, access, and practices concerning condom use in rural Mwanza region after implementation of adolescent sexual and reproductive health program (MkV).	Community-based case-control study using participant observation in 9 villages (2 multi-ethnic mining villages, 2 poor farming villages, 3 multiethnic fishing villages, 2 control villages) using 6 researchers (male and female).	Content analysis of transcripts after translation into English.	**CONDOM KNOWLEDGE/BELIEVES:**<br>- **Source of knowledge:** radio, public meetings/outreach, school, gossip; no clear understanding/ experience with condom use (access to knowledge higher in intervention communities).<br>- **Perception/misconception:** belief that condoms are neither effective nor safe; condoms might be infected with HIV; condoms are white people strategy to harm Africans through promotion and distribution of old/inferior or HIV infected products.<br>**ATTITUDES TOWARDS CONDOM USE:**<br>- **Positive attitudes:** only few girls/women, mostly pupils, valued condoms as FP method.<br>- **Negative attitudes:** condoms reduce sexual pleasure (particularly men); no decision-making power regarding condom use (particularly women, commercial sex); no need if not interested in FP; leaving exposure risk to chance/God's will; perceived low personal risk of acquiring STI/AIDS; trust in partner; fear of stigma/rejection/punishment (infidelity, promiscuity); general suspicion of condoms.<br>**CONDOM USE:**<br>- **Consistency/frequency:** majority of respondents have never used condoms; consistent use rare.<br>- **Condom use men:** only if they suspect to be at high risk of acquiring a disease.<br>- **Condom use women:** many women use only at partner's initiative; few insist on use during intercourse.<br>**CONDOM AVAILABILITY/ACCESS:**<br>- **Condom outlets: s**hops, health facilities, local promoter-distributors (intervention communities).<br>- **Stigma condom purchase:** belief health workers, salespeople, and distributors would not keep requests for condoms confidential (especially young people); customers embarrassed to request condoms.<br>- **Access:** general lack of demand; costs appear not to be a barrier; supplying rural areas and monitoring condom sales requires disproportionate amount of time and is not cost-effective.
Sonalkar et al. 2013, Kenya and Ethiopia	Perceptions regarding programmatic aspects of postpartum FP.	Cross-sectional study using phone interviews of 7 key informants (Ministry of Health officials, representatives of private organizations overseeing large FP programs) in Kenya and Ethiopia.	Content analysis.	**HEALTH SYSTEM BARRIERS:**<br>- **Planning level:** lack of consensus on definition of postpartum period; interest in postpartum IUD program; need for additional research on postpartum FP.<br>- **Supply level:** lack in quality/supply of informational materials; poor reporting of FP use in facilities; lack of supplies/poor distribution of commodities.<br>- **Human resource:** Need for health worker training; lack of skilled providers; inability to access skilled providers due to home deliveries; health extension workers to provide outreach.<br>- **Financial resources:** availability of sufficient funding for FP.

FGD = focus group discussion; FP = family planning

Authors, year, location	Study objective	Study design	Analytical approach	Main themes, thematic relationships
Ward et al. 1992, Guatemala	Sociocultural barriers to FP use among Mayans.	Community-based cross-sectional study including 9 FGD with each 8-12 participants (for women and men each 1 with singles between the ages of 15-20, each 1 with married individuals between the ages of 15-20, each 1 with married individuals between the ages of 25-30, each 1 with married individuals between the ages of 35-40, and 1 with male community leaders) and 25 in-depth interviews with TBA of same communities.	Content analysis of transcripts after translation into Spanish.	**COMMUNITY INFLUENCES:**<br>- **Approval of FP:** disapproval of those openly practicing FP; especially women suspected of using FP are severely criticized; one would have to hide FP use from friends and family; community norms dictate unmarried women to not be knowledgeable in reproduction; community leaders resistant to accept FP.<br>- **Source of FP knowledge:** Catholic Church offering premarital classes to encourage prospective couples to have all the children God sees fit; FP considered equivalent to abortion, which is murder or mortal sin.<br>- **Gender roles and FP:** men and women felt both partners should be involved FP decisions, while other family members should not; traditionally, men are decision-makers (including FP); women face difficulties discussing FP without being accused of unfaithfulness/infidelity.<br>**FP PURPOSES:**<br>- **Social norms on FP:** having children considered purpose of human existence, principal reason for marriage; traditionally, large families considered favorable, as it provides economic support in old age and strength to Mayan identity; FP use associated with laziness in fulfilling God's plan.<br>- **Women's interest in FP:** older women perceive many and closely spaced children bad for health, family finances, household workload, providing sufficient food for everyone (including breastfeeding); interest in birth spacing;<br>- **Men's interest in FP:** large families reduce size of each son's share of land; interest in birth spacing.<br>- **Knowledge of FP:** mostly traditional methods (postpartum abstinence, long periods of breastfeeding); modern FP primarily associated with birth limiting/abortion, not associated with birth spacing.<br>**MODERN FP METHODS:**<br>- **Knowledge of modern FP:** men and older women most knowledgeable about modern methods (OCP, condom, IUD)<br>- **Perception of OCP:** causes weight/loss, general debilitation, cancer, intrauterine child death, maternal death; too expensive to use.<br>- **Perception of condoms:** only used by young unmarried men; for disease prevention, not pregnancy prevention; causes female genital cancers.<br>- **Perception of IUD:** causes weight gain, maternal death.<br>**TRADITIONAL FP METHODS:**<br>- **Knowledge of traditional FP:** calendar rhythm method common, but incorrectly used; herbal, mineral, medical (intravaginal aspirin) considered as natural/traditional contraceptives/abortifacients (usually used by women who got pregnant prior to marriage); postpartum abstinence.<br>**FP SERVICE PROVISION:**<br>- **Availability:** government centers, health posts; FP information only available in Spanish; only directed to women, not to men;<br>- **Perception of community**: distrust of motives of FP promoters, especially among men.<br>- **Perception of TBA:** majority feels FP should be provided through TBA; concern that FP services are not wanted by communities.

FGD = focus group discussion; FP = family planning; TBA = traditional birth attendant(s)
